# Surface modification of probiotics at single-cell resolution: Strategies, applications, and future directions in inflammatory bowel disease

**DOI:** 10.1016/j.mtbio.2025.102485

**Published:** 2025-10-30

**Authors:** Li Peng, Xinyu Wang, Yafen Wang, Jueshuo Guo, Jianhong Yang

**Affiliations:** a Department of Pharmacy, General Hospital of Ningxia Medical University, Yinchuan, PR China; b College of Pharmaceutical, Ningxia Medical University, Yinchuan, PR China

**Keywords:** Inflammatory bowel disease, Single-cell surface modification, Probiotics, Gut microbiota, Therapeutic applications

## Abstract

Inflammatory bowel disease (IBD) manifests as a chronic immune-mediated gastrointestinal disorder is characterized by multifactorial pathogenesis involving dysregulated immune responses and intestinal dysbiosis. This pathobiological interplay results in substantial impairment of patient quality of life while elevating colorectal carcinogenesis risk. Current therapeutic strategies primarily focus on inflammatory suppression and sustained remission, yet the clinical effectiveness is compromised by nonspecific biodistribution and systemic adverse events. Emerging evidence underscores the therapeutic potential of probiotics in preventing and treating IBD. Probiotics contribute to intestinal health by restoring microbial equilibrium, enhancing gut barrier function, and mitigating inflammation. Probiotics enhance immune function and stimulate the production of short-chain fatty acids (SCFAs), essential for intestinal health. However, the effectiveness of natural probiotics is often undermined by the harsh gastrointestinal environment, including poor gastric acid survival and weak mucosal adhesion, as well as physiological factors like oxidative stress from reactive oxygen species (ROS) and variable antibiotic exposure. To overcome these challenges, single-cell surface modification strategies have been developed to enhance probiotic functionality, stability, and targeted delivery. This review provides a comprehensive summary of recent advancements in probiotic surface modification technologies. It analyzes their classification, structural design, and functional attributes, systematically comparing physical, chemical, and biological strategies. This review also discusses the benefits, limitations, challenges, and future prospects of single-cell probiotic modification.

## Graphical abstract

Single-cell modification strategies for probiotics can be categorized into physical, chemical, and biological modifications to camouflage probiotics, and introduces new functional properties, enhances probiotics at the nanoscale. Specific nanocoating confers exogenous functionalities to probiotics, such as immunomodulation, antioxidant activity, and anti-inflammatory properties, thereby enhancing the therapeutic efficacy for colitis while improving safety.

**Figure:**
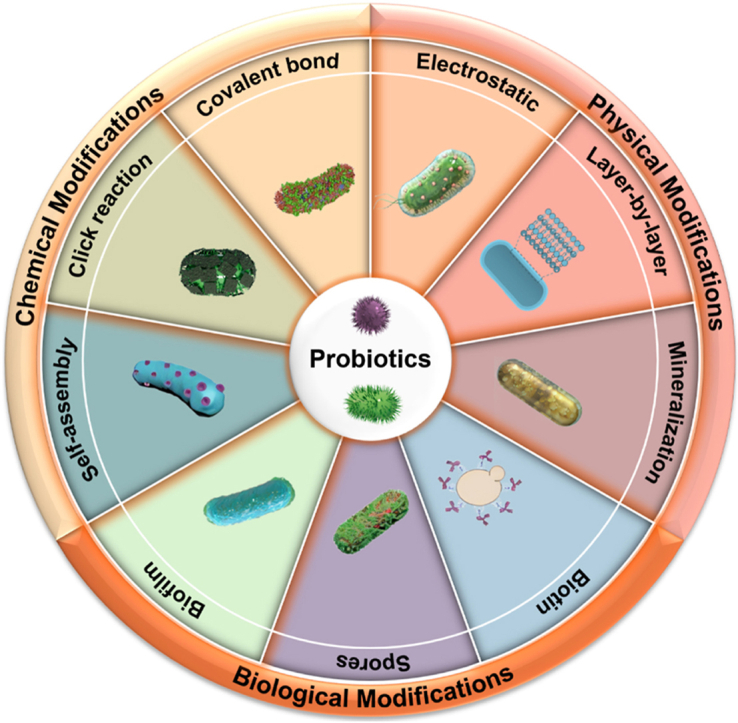

## 1 Introduction

IBD affects over 7 million worldwide, with increasing cases prevalence across all age groups, places substantial strain on health-care systems and society especially in low-income countries [1,2]. Extensive research has identified microbiota dysbiosis as a key pathogenic factor closely associated with IBD [[3], [4], [5], [6], [7], [8], [9]]. Probiotics have attracted significant interest in the treatment of IBD, due to their unique characteristics like genetic manipulation, rapid proliferation, colonization ability, and targeted host interactions in maintain intestinal homeostasis [[10], [11], [12], [13]]. Owing to the convenience of non-invasiveness and safety, oral probiotic therapy has been widely applied clinically as an adjunctive treatment [[14], [15], [16]]. However, oral probiotic delivery technologies face challenges, including chemical factors like acidic stomach conditions and bile salts that can deactivate probiotics, and physical factors like rapid gastrointestinal transit, which limits probiotic adhesion and growth in the intestines [[17], [18], [19], [20]]. Various solutions, such as nanoparticles [21], nanocrystals [22], polymer gels [23], enteric coatings [24], and patches [25], have been developed to address these challenges from chemical degradation and enhance mucoadhesion, enabling controlled release and absorption. While these methods have improved oral delivery for small molecules and some biologics, few effectively address the unique challenges of delivering live probiotics, given their large size and specific viability and growth requirements. Therefore, developing effective methods to protect probiotics from gastric acid and bile salt exposure while enhancing mucosal adhesion capabilities has become a key focus of current research efforts.

To ensure probiotics provide health benefits, it is crucial to administer sufficient viable cells. This enables them to survive the upper gastrointestinal tract (GIT) and successfully colonize the intestinal mucosa [26]. Probiotic encapsulation, an emerging technology that enhances cellular functions through surface modification, has recently advanced for probiotic applications including microencapsulation and single-cell modification [27]. Microencapsulation systems, utilizing pH-, enzyme-, redox-responsive, or pressure-triggered release mechanisms, are constructed via methods such as drying, complex coacervation, extrusion, and emulsification [17,[28], [29], [30], [31]]. However, each of these methods has limitations that restrict their broader application. For example, spray drying involves atomizing bacterial suspension into fine droplets and exposing them to hot air to rapidly evaporate water. During this process, probiotics endure multiple stresses, including heat, dehydration, shear, and osmotic stress, which can compromise cell integrity and reduce the number of viable microorganisms, thus limiting their effectiveness in probiotic formulations [32,33]. Freeze-drying, though effective, is costly and time-consuming, with potential cell damage during freezing [34]. Extrusion and emulsification offer limited protection from stomach acid and bile salts and often result in inconsistent capsule sizes [35]. Unlike traditional bulk encapsulation, which encases probiotics in larger matrices, single-cell modification is an advanced technique where individual cells are isolated and surrounded by a protective matrix, offering individualized protection to each cell [36]. Single-cell encapsulation boosts the stability and viability of probiotics by providing a protective barrier against harsh conditions and oxidative stress [18]. This method ensures probiotics survive the stomach's acidity, reach the intestines in a viable state, and deliver their benefits. It also enables more precise and controlled release at specific sites within the GIT, responding to triggers like pH changes, enzymes, or redox conditions, thus enhancing probiotic therapy efficacy. Additionally, single-cell encapsulation prolongs the shelf life of probiotic products by preserving cell viability during storage and transport.

The bacterial membrane is composed of various substances such as peptidoglycan, teichoic acid, lipopolysaccharide, and proteins. Its surface exhibits a negative charge and possesses multiple functional groups including hydroxyl, carboxyl, and amino groups [37]. These common molecules provide convenient avenues for surface modification of bacteria through physical and chemical methods such as covalent coupling, electrostatic interactions, and hydrophobic interactions, to ensure the functional modification, protection, and delivery of live bacteria [38]. These single-cell modification systems, developed through physical, chemical, and biological methods, offer new possibilities for the precise delivery of probiotics. This study offers a systematic review of single-cell surface modification techniques for probiotics used in IBD and discusses the limitations and prospects of these methods.

## 2 Classification and composition of the single cell probiotic modification system

**Fig. 1:**
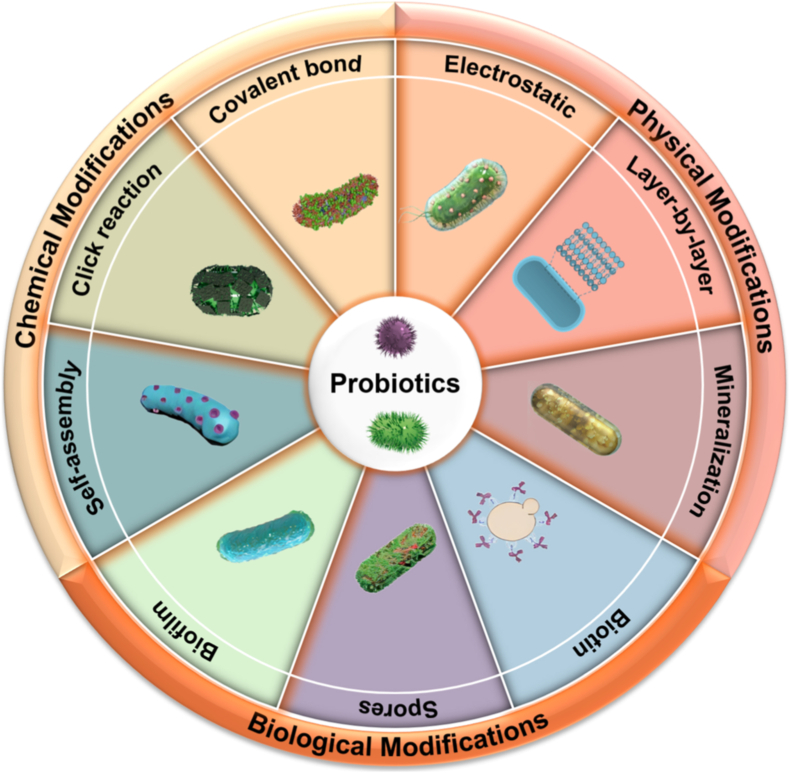
Schematic illustration of common strategies of single-cell modification systems for probiotics.

Single-cell modification technique employs surface encapsulation to camouflage probiotics, providing protection and introducing new functional properties. The nanoscale enhancement enables direct delivery to the colon without release from the encapsulating matrix [39]. These strategies significantly improve probiotic protection in complex GIT environments, enhance mucin adhesion, and reduce the risk of bacterial translocation. Additionally, specific nanocoating imparts exogenous functions such as immunomodulation, antioxidant activity, and anti-inflammatory properties, enhancing the efficacy and safety of IBD treatment [27,39,40]. Single-cell probiotic modification can be classified into physical, chemical, and biological modifications. Based on material properties and the surface characteristics of probiotics, various mechanisms such as electrostatic adsorption, covalent and non-covalent binding, chemical crosslinking, co-deposition, physical extrusion, click reactions, and others are utilized to develop protective and functional coatings (Fig. 1).

**Fig. 2:**
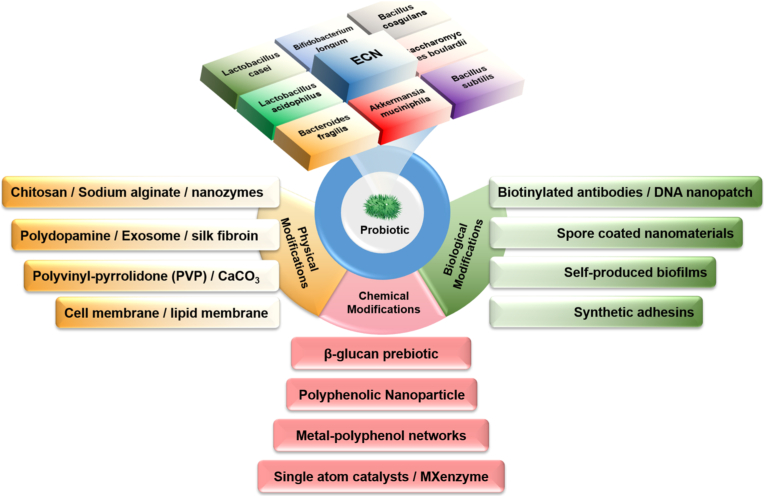
Schematic illustration of the most common composition single-cell modification systems. Common probiotics selection, varied modification strategies, various enteric polymers, metal-polyphenol network (MPN), lipids and bio-membrane, nanozymes, biotinylated antibodies, and combination of multiple materials.

Probiotics inherently possess surface antigens, adhesion factors, and flagella that play a crucial role in their interactions with the surrounding environment and communication with other probiotics [27]. By employing material chemistry, the surface of probiotics can be modified to create coatings that impart new exogenous functions [41,42]. For example, *Escherichia coli* Nissle 1917 (EcN), a Gram-negative probiotic strain, exhibits substantial therapeutic potential in treating intestinal disorders, is known for its strong anti-inflammatory, and intestinal microbiota-regulating properties [43,44]. It is a key component of Mutaflor®, a medication used in the treatment of IBD [45]. Materials like alginate (AG) and chitosan (CS) [46], mesoporous silica [47], carbon quantum dots (CQDs) [48], liposomes [49], silk fibroin [50], and tannic acid (TA) et cetera, have been used to decorate probiotics, offering protection against physiological gastrointestinal conditions and improving therapeutic efficacy. The common single-cell modification probiotics and materials are shown in Fig. 2.

## 3 Single-cell surface modification technologies for probiotics

**Fig. 3:**
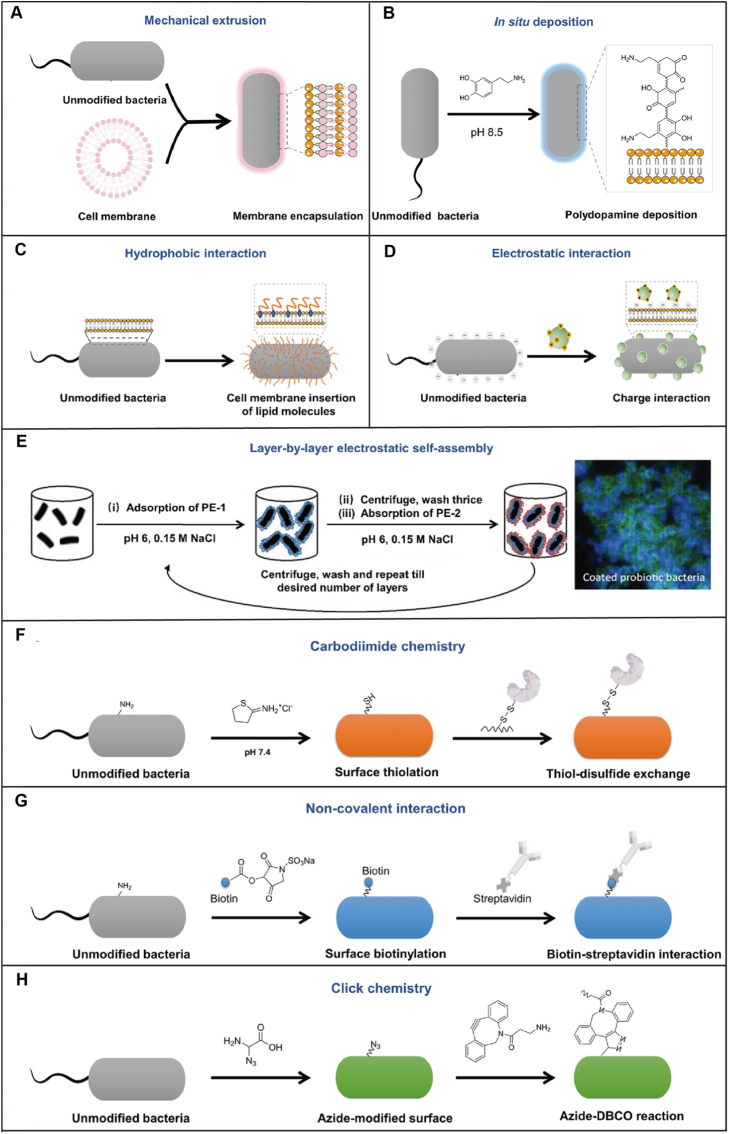
Strategies for single-cell modification of probiotics [10]**. A)** Coating bacteria with cell membranes by mechanical extrusion. **B)** Coating bacteria with cell membranes through the generation of apoptotic bodies. **C)** Insertion of hydrophobic lipid molecules into the lipid layer of bacterial outer membrane by a hydrophobic interaction. **D)** Absorption of positively charged molecules on negatively charged bacterial surface via electrostatic interaction. **E)** Coated bacteria with multiple layers through alternative adsorption of oppositely charged polyelectrolytes on bacterial surface. The first layer (blue) and the second layer (red) separately indicate polyelectrolytes (negative charge) and chitosan (positive charge). **F)** Conversion of the amino groups on bacterial surface to free thiols via a one-step imido ester reaction and the formation of disulfide bonds between modified bacteria and poly(disulfide)s-enriched mucin by dynamic thioldisulfide exchange reaction. **G)** Decorating bacterial surface with biotin by using sulfa-NHS-biotin and the attachment of various streptavidin-derived substances via specific biotin-streptavidin interaction. **H)** Anchoring azide groups (-N_3_) on bacterial surface via D-amino acid metabolism and subsequent conjugation of DBCO-tagged molecules by a click reaction.

The cell surface of a microorganism is negatively charged due to teichoic acids and peptidoglycan, allowing positively charged functional materials to adhere via electrostatic attraction thus encapsulating the microorganisms [51]. The charge status, chemical groups, proteins, and antigens on the bacterial surface directly influence their physiological functions, such as adhesion, proliferation, and differentiation. Once the bacterial surface is altered, specific physiological signals and responses can be triggered. Surface modification generally aims to avoid potential side effects of bacteria while enhancing their functionalities and applications. This is achieved through variations in surface characteristics and subsequent interface interactions. As our understanding of bacterial surface properties deepens and interdisciplinary knowledge converges, various physical, chemical and biological techniques have emerged as effective methods for bacterial surface decoration (Fig. 3). Table 1 summarizes versatile single-probiotic modification systems that utilize single-cell coating technology. It outlines the probiotic species, coating material classifications, modification methods, and functional applications, all of which will be discussed in detail below.

**Table 1:** Overview of different strategies, probiotic strains, principles, materials, and functions of single-cell surface modification.

Strategy	Principle	Probiotic	Material	Function	Reference
Physical modifications	Electrostatic interaction	*Bacillus coagulans*	Polysaccharide CS	• Improved the survival of probiotics *in vitro* and *in vivo* (3-times) • Improved adhesion in intestinal tissue (6-times)	[46]
	Electrostatic interaction	EcN	5-ASA and sodium AG	• Improved the survival of probiotics *in vitro* and *in vivo* • PH-responsive release	[59]
	Electrostatic interaction	EcN	CaCl_2_, CS and AG	• Improved the survival of probiotics *in vitro* and *in vivo* • Improved adhesion in intestinal tissue	[61]
	Electrostatic interaction	EcN	Glycosylated CS and sodium AG	• Improved the survival of probiotics *in vitro* and *in vivo* (100-times) • Improved adhesion in intestinal tissue (300-times)	[56]
	LbL	*Saccharomyces boulardi*i	Polyelectrolytes, CA, and dextran sulfate	• Improved the survival of probiotics *in vitro* and *in vivo*	[62]
	Electrostatic interaction	EcN	CS and AG	• Improved the survival of probiotics *in vitro* and *in vivo* (5-times) • Improved adhesion in intestinal tissue (4-times)	[58]
	Electrostatic interaction	EcN	Hydrophobically modified hydrogen silicate nanosheets	• Improved the survival of probiotics *in vitro* and *in vivo* (100-times) • Synergistic therapeutic effect on UC	[47]
	Self-assembly	EcN	Medicative SF	• Improved the survival of probiotics *in vitro* and *in vivo* (52-times) • Improved mucoadhesion in intestinal tissue (5.8-times higher colonization) • Synergistic therapeutic effect on UC	[50]
	Electrostatic interaction	*Lactobacillus rhamnosus* GG	Balsalazide and liposomes	• Improved the survival of probiotics *in vitro* and *in vivo* (1.6-times) • Improved adhesion in intestinal tissue • Synergistic therapeutic effect on UC	[74]
	Interfacial Electrostatic interaction	EcN	CS-modified epigallocatechin gallate and gold nanozymes	• Improved the survival of probiotics *in vitro* and *in vivo* (After 2 h incubatuion with SGF, more than 1400 viable probiotics persisted in the ECA@EcN group, while nearly all uncoated EcN cells were eliminated) • Improved adhesion in intestinal tissue (4.5-times) • Synergistic therapeutic effect on UC	[75]
	Electrostatic interaction	EcN	Fe^3+^ modified montmorillonite	• Improved the survival of probiotics *in vitro* and *in vivo* • Improved adhesion in intestinal tissue (22.6-times increase in colonization efficiency) • Synergistic therapeutic effect on UC	[76]
	Electrostatic interaction	*Bacteroides fragilis/*EcN*/Bifidobacteria*	Polyvinyl-pyrrolidone CaCO_3_	• Improved the survival of probiotics *in vitro* and *in vivo* (6-times) • PH-responsive release	[69]
	Electrostatic interaction	*Lactobacillus rhamnosus* GG	NLRP12 plasmids iMXene	• Improved the survival of probiotics *in vitro* and *in vivo* • Synergistic therapeutic effect on UC	[65]
	Electrostatic interaction	EcN	Bio-catalyzed hydrated iron nanoparticles Alginate CS	• Improved the survival of probiotics *in vitro* and *in vivo* (100-times) • Improved mucoadhesion in intestinal tissue (2.42-times) • Synergistic therapeutic effect on UC	[68]
	Electrostatic interaction	EcN *Staphylococcus aureus*	Dioleoylphosphatydic acid and cholesterol	• Improved the survival of probiotics *in vitro* and *in vivo* (3-times higher in the mouse stomach and more than 4 four-times higher bioavailability in the gut)	[49]
Chemical modifications	Electrostatic interaction	EcN	Polyphenolic Nanoparticle and sodium alginate	• Improved the survival of probiotics *in vitro* and *in vivo* • Improved mucoadhesion in intestinal tissue (1.30, 1.21, and 1.14-timesat 2, 6, and 24 h after oral administration) • Synergistic therapeutic effect on UC	[160]
	Electrostatic interaction	EcN	HA-functionalized MPN	• Improved the survival of probiotics *in vitro* and *in vivo* (8-times) • Improved adhesion in intestinal tissue (122.1-times) • Synergistic therapeutic effect on UC	[89]
	Imidoester reaction	ECN	Surface thiolation	• Improved mucoadhesion in intestinal tissue (170-fold higher attachment)	[80]
	LbL	EcN	TA and mucin	• Improved the survival of probiotics *in vitro* and *in vivo* (5-times) • Improved mucoadhesion in intestinal tissue • Synergistic therapeutic effect on UC	[91]
	Amidation	EcN	Ginger-derived exosome-like nanoparticles	• Improved the survival of probiotics *in vitro* and *in vivo* (21-times higher uptake efficiency) • Improved proliferation of ECN	[71]
	Amidation	EcN	IL-6 aptamers	• Improved the survival of probiotics *in vitro* and *in vivo* (Intestines: 6.9-times higher, cecum: 123.5-times higher, colon:197.2-times higher) • Improved adhesion in intestinal tissue • Synergistic therapeutic effect on UC	[108]
	Electrostatic interaction	EcN	m-PEG-NH_2_ conjugated PDA	• Improved adhesion in intestinal tissue (3-times)	[106]
	Electrostatic interaction	*Lactobacillus acidophilus*	Tungsten ion-loaded mesoporous PDA	• Improved the survival of probiotics *in vitro* and *in vivo* (1.3-times) • Synergistic therapeutic effect on UC	[161]
	Phenylboronic acid click reaction	*Lactobacillus fermentum*	Melanin Nanoparticle	• Improved the survival of probiotics *in vitro* and *in vivo* • Improved mucoadhesion in intestinal tissue • Synergistic therapeutic effect on UC	[115]
	Click reaction	*Lactobacillus longum*	Single-atom catalysts	• Improved the survival of probiotics *in vitro* and *in vivo* • Synergistic therapeutic effect on UC	[113]
	Click reaction	*Akkermansia muciniphila*	V2C MXenzyme	• Improved the survival of probiotics *in vitro* and *in vivo* • Synergistic therapeutic effect on UC	[114]
	Click reaction	*Lactobacillus reuteri* DSM 17938	Azido (N_3_)-modified Prussian blue nanozyme	• Improved mucoadhesion in intestinal tissue (4.5-times) • Synergistic therapeutic effect on UC	[119]
	Click reaction	ECN	C18-PEG-PBA linked Zn and I3C	• Improved mucoadhesion in intestinal tissue • Synergistic therapeutic effect on UC	[116]
	Covalent bonds	ECN	TA and iron ions	• Improved the survival of probiotics *in vitro* and *in vivo*	[87]
	Click-chemistry	*Bifidobacterium infantis*	Pathogen-derived CQDs and mesoporous nanoparticles	• Improved the survival of probiotics *in vitro*	[48]
	Covalent bonds and hydrogen bonds	*Lactobacillus rhamnosus*	Tea polyphenols	• Improved the survival of probiotics *in vitro* and *in vivo* • Improved mucoadhesion in intestinal tissue (2.67-times) • Synergistic therapeutic effect on UC	[96]
	Covalent bonds and hydrogen bonds	EcN	PDA	• Improved the survival of probiotics *in vitro* and *in vivo* (5–10 times)	[98]
	Covalent bonds and hydrogen bonds	EcN	CS and PDA	• Improved the survival of probiotics *in vitro* and *in vivo* (30-times) • Improved mucoadhesion in intestinal tissue (4-times)	[99]
	Amidation	*Bifidobacterium longum*	Hafnium disulfide nanosheet and HA-bilirubin	• Improved mucoadhesion in intestinal tissue • Synergistic therapeutic effect on UC	[110]
	Covalent bonds and hydrogen bonds	EcN	MPN and mucin/polyoxamers 188/β-glucan	• Improved the survival of probiotics *in vitro* and *in vivo* (1720-times in SGF) • Synergistic therapeutic effect on UC	[85]
	Self-assembly	*Lactobacillus acidophilus*	Phenylboronic acid grafted DA with mucosal adhesive properties to obtain DP and HA	• Improved the survival of probiotics *in vitro* and *in vivo* • Improved mucoadhesion in intestinal tissue (7.5-times) • Synergistic therapeutic effect on UC	[77]
	Covalent bonds	EcN	HA-poly propylene sulfone and norepinephrine	• Improved the survival of probiotics *in vitro* and *in vivo* • Improved mucoadhesion in intestinal tissue (7-times) • Synergistic therapeutic effect on UC	[105]
	Self-assembly	EcN	TA and poloxamer 188	• Improved the survival of probiotics *in vitro* and *in vivo* (2.8-times high exposure to the H_2_O_2_ in 3h) • Improved mucoadhesion in intestinal tissue • Synergistic therapeutic effect on UC	[97]
Biological modifications	Bioorthogonal chemistry	*Lactobacillus plantarum*	β-glucan prebiotic	• Improved the survival of probiotics *in vitro* and *in vivo* (276-times *in vitro*, 17.27-times *in vivo*) • Synergistic therapeutic effect on UC • Colon-targeted degradation	[129]
	Maltodextrin transporter pathway	EcN	DNA nanopatch CS and AG	• Improved the survival of probiotics *in vitro* and *in vivo* • Synergistic therapeutic effect on UC	[137]
	Self-coated	*Bacillus subtilis*	Self-produced biofilms	• Improved the survival of probiotics *in vitro* and *in vivo* • Improved mucoadhesion in intestinal tissue	[141]
	Electrostatic interaction	*Lactobacillus casei*	Pericellular film Selenium dot	• Improved the survival of probiotics *in vitro* and *in vivo* • Improved adhesion in intestinal tissue	[144]
	Catechol chemistry	*Lactobacillus*	Phenolic compounds	• Improved the survival of probiotics *in vitro* and *in vivo* (1.4 times) • Improved mucoadhesion in intestinal tissue	[145]
	Physical extrusion	*Bacillus*	Spore coat nanomaterial	• Improved the survival of probiotics *in vitro* and *in vivo* • Improved mucoadhesion in intestinal tissue	[132]
	Esteramine chemistry	*Lactobacillus*	Synthetic adhesins	• Improved mucoadhesion in intestinal tissue	[149]
	Biotin-streptavidin interaction	ECN	Poly(methyl methacrylate) functionalized streptavidin	• Improved mucoadhesion in intestinal tissue (10-times)	[148]
	Biotin-streptavidin bond	*Saccharomyces boulardii*	Biotinylated antibodies	• Improved mucoadhesion in intestinal tissue (350-times higher binding capability with relevant ECM proteins compared to non-targeted strains and maintained at least 15-times higher binding capability after 48 h in a simulated gastrointestinal environment.)	[154]
	Electrostatic and hydrogen bonding interactions Genetic engineering	EcN	Oxidized starch and polyethylene imine	• Improved the survival of probiotics *in vitro* and in *vivo* (40-times in stomach and 74-times in small intestine)	[157]
	Genetic engineering	ECN	Plasmid of tyrosinase gene	• Improved the survival of probiotics *in vitro* and *in vivo* • Improved adhesion in intestinal tissue (4.5-times) • Synergistic therapeutic effect on UC	[158]

### 3.1 Physical modifications

Physical modification utilizes techniques, including electrostatic interactions and physical extrusion, to coat probiotics with functional materials. Surface modification can prolong the initial growth phase of probiotics due to their need for nutrient permeability, although it does not impact the overall growth potential. This growth capability depends on the number of coating layers, the modification process, and the composition of the shell materials [52].

#### 3.1.1 Layer-by-layer (LbL) assembly

**Fig. 4:**
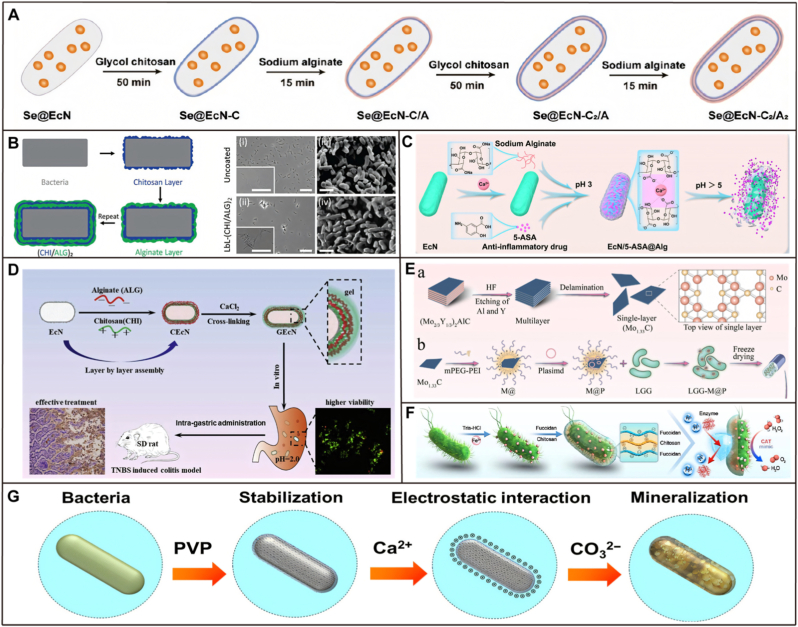
Surface physical modification of probiotics. A) Preparation and characterization of Se@EcN-C_2_/A_2_ [56]. **B)** LbL modification of probiotics. a) Schematic LbL templating of CS and AG on probiotic. b) Brightfield images of (i) uncoated-BC and (ii) LbL-(CHI/ALG)_2_-BC. SEM images of (iii) uncoated-BC and (iv) LbL-(CHI/ALG)_2_-BC [46]. **C)** Schematic illustration of (a) the nanoencapsulation of probiotics and an anti-inflammatory agent, 5-ASA, by a gastrointestinal microenvironment responsive alginate coating [59]. **D)** Schematic Description of the GE@Cn and Its Role in Treating IBD [61]. **E)** Schematic illustration of the LGG-M@P cascade bi-enzymatic system for repairing inflammatory bowel disease lesions. a) Etching and intercalation process of (Mo_2/3_Y_1/3_)_2_AlC. b) Synthesis procedure of LGG-M@P cascade bi-enzymatic system [65]. **F)** Schematic illustration of the development of engineered probiotics for multipronged management of IBD and the characterization of EcN-Fh [68]. **G)** Schematic illustration of biointerface mineralization that generates ultraresistant gut microbes as oral biotherapeutics. Preparation of mineral coating on bacterial surface [69].

Among these strategies, LbL assembly technology effectively encapsulates probiotics by alternately depositing polymers with opposite charges, directly promoting the adhesion, growth, and proliferation of modified probiotic on the intestinal surface. Sodium AG and CS have become mainstream embedding materials due to their excellent biocompatibility, degradability, and adhesiveness [53]. CS carries a positive charge and interacts with intestinal mucins through electrostatic attraction, hydrogen bonding, and hydrophobic effects [54]; Sodium AG is an anionic polymer rich in carboxyl groups, which adheres to the mucosa via hydrogen bonding [55]. In the LbL self-assembly modification process, they are designed as probiotic nanofilms. Upon oral administration, they undergo degradation in the intestine, thereby exhibiting their adhesive properties and enabling sustained probiotic colonization. For example, Li et al. [56] developed a “three-in-one” probiotic for the treatment of UC. The researchers used the clinically approved probiotic EcN as a carrier and employed biomineralization technology to synthesize selenium (Se) nanoparticles within the bacterial cells, forming Se@EcN. Subsequently, they coated the surface of Se@EcN with layers of glycosylated CS (C_2_) and sodium AG (A_2_) to create a dual protective structure, resulting in Se@EcN-C_2_/A_2_. This enhanced its gastrointestinal stability and targeting capability at inflammation sites (Fig. 4A). Additionally, the synergistic effect of sodium AG and CS compensates for the solubility defect of CS in gastric acid and the porous structure of sodium AG. Its pH sensitivity maintains structural integrity in gastric acid, disintegrating in the intestine to release probiotics and act as prebiotics [57]. As a positively charged cationic polysaccharide, CS could form an electrolyte composite gel with anionic sodium AG. LbL modification of *Bacillus coagulans* (BC) significantly enhances its acid-cholate resistance, adhesion, and survival rate [46] (Fig. 4B). Zhou et al. [58] engineered EcN (ECN-pE) to overexpress catalase and superoxide dismutase for treating intestinal inflammation. To enhance the gastrointestinal bioavailability, ECN-pE was coated with biofilm-forming polymers CS and sodium AG using LbL electrostatic assembly. This double-layer armor significantly improved the survival rate of EcN in the two simulated solutions, maintaining over 10^4^ colony forming unit (CFU)/mL within 2 h. Additionally, the armor enhanced the adhesion of EcN to the intestine. *In vivo* images indicating a higher survival rate and better intestinal retention. Similarly, Peng et al. [59] introduced a straightforward and secure method for UC treatment by encapsulating probiotics and the anti-inflammatory drug 5-aminosalicylic acid (5-ASA) in alginate polysaccharides responsive to the gastrointestinal microenvironment. The AG-based coating, resistant to acid, safeguards probiotics from the harsh gastric conditions. Once in the intestines, the coating degrades in environments with a pH above 5, releasing the probiotics and 5-ASA (Fig. 4C). The polysaccharide structure of alginate, when combined with calcium ions, forms a protective colloidal framework around probiotics, safeguarding them from gastric fluid after oral administration [60]. Upon reaching the intestines, the alginate coating dissolves at neutral pH, releasing the probiotics and therapeutic drugs to enhance the treatment of intestinal diseases [53]. The cross-linking principle of sodium AG with calcium ions is based on electrostatic interactions, where the carboxyl groups in sodium alginate form ionic bonds with calcium ions, thereby creating a three-dimensional network structure of gel. Luo et al. [61] enhanced the stability of encapsulated EcN by cross-linking the outer layer of sodium AG with calcium ions, improving its survival rate in the GIT and alleviating 2,4,6-trinitrobenzenesulfonic acid (TNBS) induced UC (Fig. 4D). Midhun Ben Thomas et al. [62] encapsulated *Saccharomyces boulardii* using the LbL with oppositely charged polyelectrolytes, CA, and dextran sulfate, to protect it from degradation during gastrointestinal transit. This coating improved cell viability after lyophilization and exposure to simulated gastrointestinal conditions. This study highlighted the selective permeability of coated cells playing a crucial role in maintaining the integrity and vitality of yeast cells.

In an environment rich in calcium ions, bacteria form a stable lipid layer on their surfaces through the electrostatic interactions between calcium ions and amphiphilic phospholipids, specifically dioleoyl phosphatidic acid [49,63]. Similarly, surface anchoring by calcium ions enables the enteric polymer Eudragit L100-55 to self-assemble on the bacterial surface via ionic bonds, thereby forming a pH-responsive smart coating [64]. By engineering probiotics followed by LbL modification, additional functionalities can be conferred to probiotics, further ensuring their bioavailability at disease sites. However, this approach does not account for the impact of high ROS levels in UC inflammatory environments on probiotics. Researchers have embedded nano-catalysts with high ROS scavenging capabilities into the lipid layer, forming composite coatings with antioxidant, stability, and biocompatibility. Such coatings substantially improve probiotic survival rates in the GIT and alleviate UC symptoms through ROS scavenging activity. Yu et al. [65] developed a probiotic bi-enzymatic cascade system to modulate the intestinal environment and treat IBD. The system employs iMXene (M), which scavenges intestinal ROS and utilizes its large surface area to deliver dCas9-upregulated NLRP12 plasmids (P), forming M@P. This complex is electrostatically adsorbed onto live *Lactobacillus rhamnosus* GG (LGG), creating the LGG-M@P system, which maintains LGG's viability and iMXene's enzymatic functions. After freeze-drying, the bi-enzymatic LGG M@P is encapsulated in enteric-coated capsules to ensure intestinal delivery (Fig. 4E).

#### 3.1.2 Biomineralization

Biomineralization is an effective strategy for organisms to biologically camouflage themselves that involves forming a mineral-like layer on the probiotic surface to protect it from damage by gastrointestinal environments [66]. Organisms use biomineralization to create a hard coating around their soft surface, improving survival by providing support and protection [67]. This process involves the directed deposition of minerals either within bacterial cells, such as iron oxides and sulfides in magnetotactic bacteria, or on their surfaces. Bio-interface mineralization technology is another current probiotic surface modification strategy. For example, Chen et al. [68] proposed a probiotic-safe in situ mineralization method, which involves the in-situ growth of bio-catalyzed hydrated iron nanoparticles (Fh NPs) on probiotics through a one-step bio-stimulated mineralization process. The probiotics modified with Fh NPs are further encapsulated with a protective shield derived from ulvan, enhancing colonic delivery. Ulvan is a specific ligand for P-selectin protein, which is overexpressed in inflamed intestines, and it also promotes electrostatic binding with positively charged proteins accumulated in damaged intestinal epithelium (Fig. 4F). Geng et al. [69] used polyvinylpyrrolidone-mediated bio-interface mineralization technology to coat probiotics with a calcium carbonate outer layer. Results showed that adding calcium ions and carbonate ions sequentially to a probiotic dispersion can form a calcium carbonate deposition coating within a short time. This method is applicable to various bacterial strains with high modification efficiency. The coating shields probiotics from oxygen, UV light, and alcohol during production and storage. It enables quick neutralization of gastric acid for the smart release of bacteria. Released calcium ions help aggregate bile acids, protecting probiotics from damage by gastric acid and bile acids, thus enhancing oral bioavailability (Fig. 4G).

#### 3.1.3 Silk fibroin

**Fig. 5:**
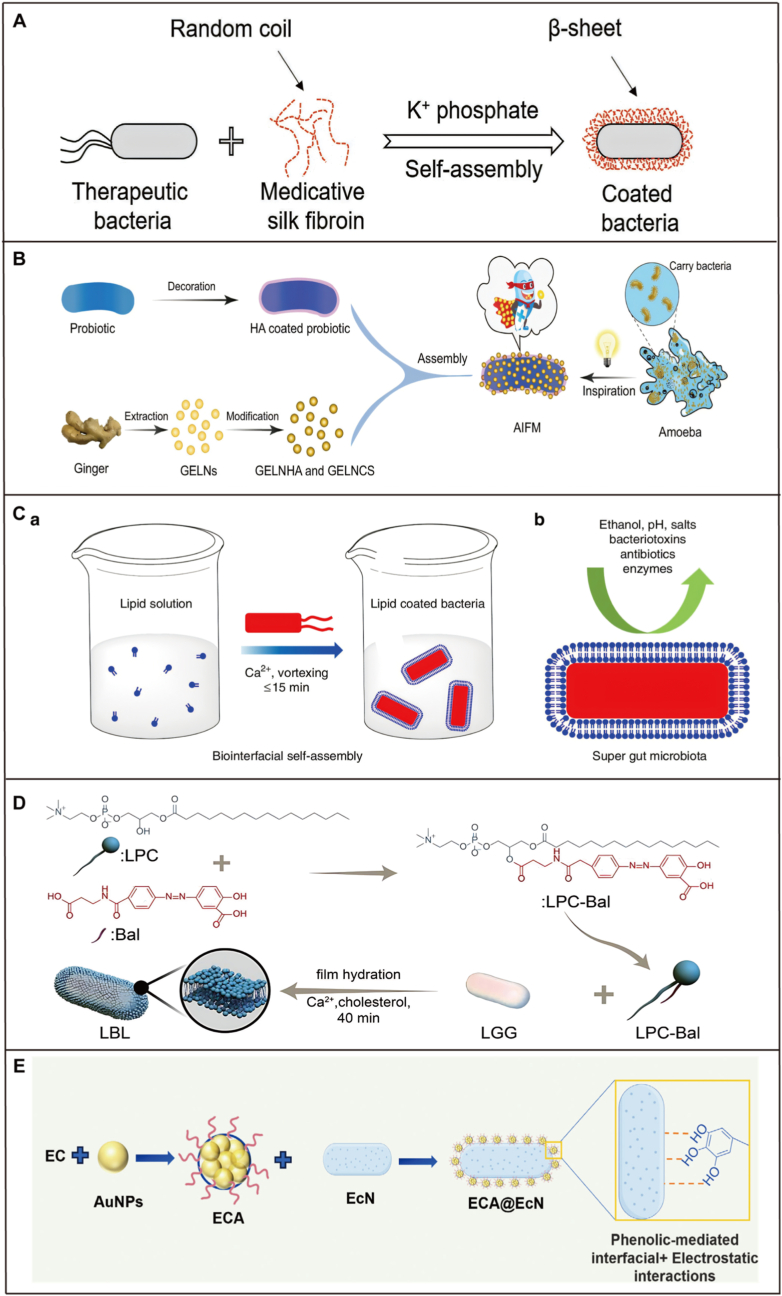
Surface physical modification of probiotics. A) Coating therapeutic bacteria with medicative silk fibroin by biointerfacial self-assembly [50]. **B)** The construction procedures of AIFM [71]. **C)** Preparation and characterization of LCB. a: Schematic illustration of the preparation of lipid membrane coated bacteria by biointerfacial supramolecular self-assembly. b: The presence of coating membranes endows probiotic bacteria with exceptional resistance to various harsh environmental conditions [49]. **D)** Schematic illustration of synthesis and the structure of LPC-Bal [74]. **E)** Schematic illustration of the construction of ECA@EcN [75].

Silk fibroin (SF) makes up 70–80 % of the natural protein polymers in silk from the *Bombyx mori* cocoon, protecting pupae from harsh environmental conditions. It is rich in anti-parallel β-sheet structures, creating a strong network [70]. This β-sheet network provides defense against acid and bile stress on the cell surface and transitioning from a random coil to a β-sheet conformation can self-assemble on bacterial surfaces. Hou et al. [50] detail a method to enhance probiotics using a SF nanocoating, inspired by the protective and therapeutic properties of silkworm cocoons. SF possesses anti-inflammatory properties, achieves self-assembly on the surface of bacteria through the electrostatic interactions of potassium ions in potassium phosphate solution, accompanied by conformational changes of the SF during this process. This preparation avoids organic solvents and chemical reagents, minimizing impact on bacterial viability. The SF nanocoating boosts bacterial survival in simulated gastric fluid by nearly 52 times compared to uncoated bacteria. After oral administration, coated bacteria achieve 5.8 times higher colonization in the mouse intestinal tract than uncoated bacteria. Additionally, SF nanocoating enhances the therapeutic effect of probiotics in a murine model of intestinal mucositis (Fig. 5A).

#### 3.1.4 Hydrogen bonds and covalent bonds

Nutrient competition with indigenous microbes or pathogens presents a significant challenge for oral probiotic efficacy. To address this issue, Pan et al. [71] developed an amoeba-inspired food-carrying strategy (AIFS) by prebinding ginger-derived exotics like nanoparticles (GELNs) onto probiotics as nutrient depots. AIFS enables probiotics to exclusively and efficiently consume GELNs in situ, even among competing bacteria, achieving up to 21-times higher uptake efficiency than unmodified probiotics and dramatically accelerating their proliferation (Fig. 5B). In combination with co-delivered drugs, engineered probiotics produce synergistic therapeutic effects, functioning as multifaceted platforms that serve as targeted drug delivery vehicles, local microenvironment modulators, and immune adjuvants.

#### 3.1.5 Biomimetic membrane

Biomimetic materials such as cell membranes are also used for single-cell modification of probiotics [72,73]. For instance, lipid coating surface modification strategies for probiotics have been reported, simplifying preparation processes without imparting extra functionalities. Liu et al. [49] developed a simple and efficient method has been reported for encapsulating bacteria into biocompatible liposomes via bio-interface supramolecular self-assembly within 15 min, significantly enhancing intestinal bioavailability and environmental resistance of the bacteria. In mouse trials, the bioavailability of the encapsulated bacteria was found to be more than 4 times higher compared to non-encapsulated bacteria. Additionally, the survival capability of the bacteria under various extreme conditions, including environments with strong acids and bases, antibiotics, and ethanol, was markedly improved. Ultimately, the liposomes disintegrate after completing their mission, allowing the bacteria to target and migrate to disease sites (Fig. 5C).

#### 3.1.6 Electrostatic interaction

Metal cations can promote surface modification by neutralizing the charge on microbial surfaces. Current studies indicate that this method involves a simple modification process with minimal impact on the intrinsic properties of probiotics. Zhang et al. [74] assembled the prodrug balsalazide (Bal) for treating UC onto liposomes composed of 1-palmitoyl-sn-glycero-3-phosphocholine (LPC), forming the prodrug nanomolecule LPC-Bal. They further modified LPC-Bal onto the surface of *Lactobacillus rhamnosus* GG (LGG), constructing prodrug-encapsulated LbL. The bioengineered probiotic coating integrates gut microbes’ protection, colon-targeted drug release, prolonged drug retention, and inflammation regulation (Fig. 5D). Self-assembly is performed on a wide range of materials including ion-organic complexes and nanoparticles, demonstrating an extremely rapid and controlled technique for producing structurally functional diverse coating on organisms. Zhu et al. [75] synthesized a CS-modified epigallocatechin gallate (EGCG-CS, EC), leveraging the intrinsic adhesive and coordination properties of polyphenols to capture gold nanozymes (AuNPs), forming ECA complexes that enhance nanozyme activity. When coated onto EcN, the resulting ECA@EcN system effectively scavenged ROS, improving probiotic viability and promoting colon accumulation (Fig. 5E). In existing research, researchers have achieved surface modification of probiotics by reacting them with nanomaterials. Yang et al. [76] developed a Fe^3+^ modified montmorillonite (MMT)-armed probiotic ECN (MMT-Fe@EcN), which enhances intestinal colonization and hydrogen sulfide (H_2_S) scavenging for synergistic alleviation of IBD. The montmorillonite layer protects EcN from environmental assaults during oral delivery and improves on-site colonization in the mucus-depleted intestinal segment due to its strong adhesive capability and electronegativity, resulting in 22.6-times increase in colonization efficiency compared to EcN alone. Additionally, MMT-Fe@EcN manages inflammation by scavenging H_2_S, thereby enhancing probiotic viability and colonization to restore the gut microbiota. Huang et al. [77] introduces a stimulus-responsive mucosal adhesive probiotic capable of specifically adhering to intestinal inflammatory sites, eliminating high levels of ROS, and regulating gut microbiota homeostasis. Initially, phenylboronic acid (PA) was grafted onto DA with mucosal adhesive properties to obtain DP. Subsequently, hyaluronic acid (HA) was reacted with DP to synthesize HDP. Finally, *Lactobacillus acidophilus* was selected, and a stimulus-responsive adhesive probiotic (Lac@HDP) was obtained. By consuming large amounts of ROS, the boronic acid groups of Lac@HDP were oxidized and cleaved, thereby exposing catechol hydroxyl groups with mucosal adhesion capabilities, significantly prolonging the retention time of Lac in inflamed intestines.

### 3.2 Chemical modifications

The process of attaching functional materials to probiotics through chemical methods is referred to as chemical modification. Beyond adding specific functional motifs, multiple chemical modifications can simultaneously equip bacteria with diverse functions or combine the essential traits of safe and effective bioagents [40,50]. The surface of probiotics contains various functional groups, such as amino, carboxyl, thiol, and hydroxyl groups, derived from components like peptidoglycan, polysaccharides, proteins, and lipopolysaccharides [78]. Consequently, chemical reactions involving these functional groups occur when microbes interact with functional materials. Chemical modification offers a flexible and versatile strategy to introduce otherwise unattainable functional structures, providing an alternative approach for creating functionalized bacterial bioagents [79,80].

#### 3.2.1 Metal-polyphenol network

**Fig. 6:**
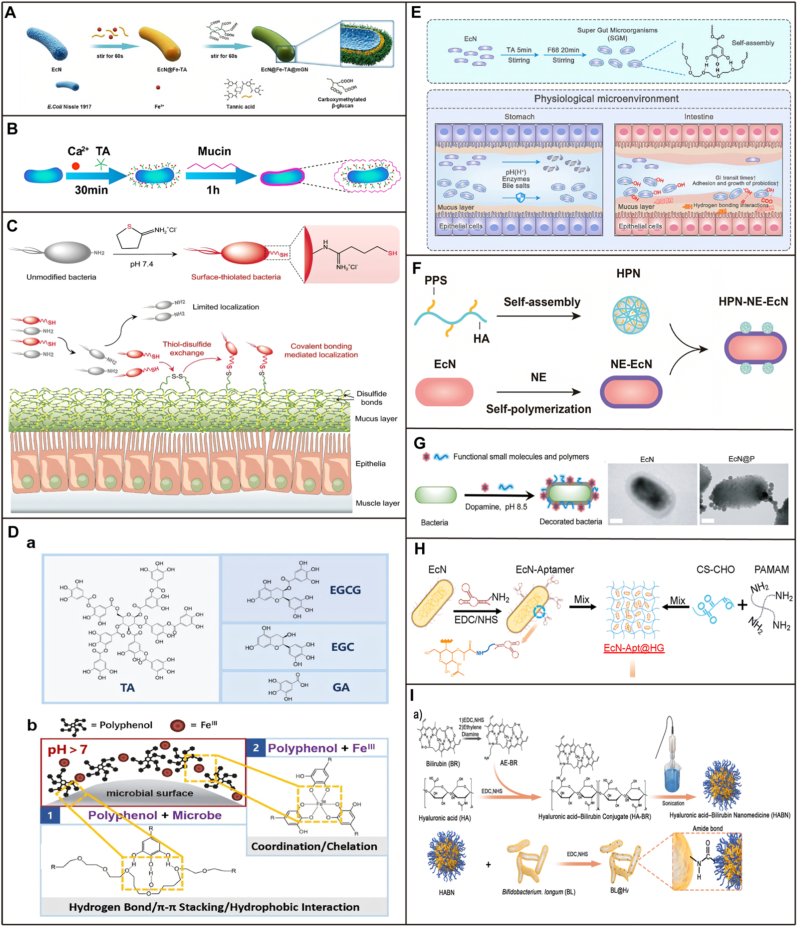
Surface chemical modification of probiotics. A) Preparation of EcN@Fe-TA@mGN [85]. **B)** Decorating the therapeutic bacteria with TA and mucin by LbL coating technology [91]. **C)** Preparation of surface-thiolated bacteria by a simple one-step imidoester reaction under cytocompatible conditions. Bonding of chemically reactive surface-thiolated bacteria with poly(disulfide)s-abundant mucin locating at various tissue interfaces by catalyst-free dynamic thiol-disulfide exchange reaction [80]. **D)** Schematic illustration of the fabrication of MPN@L [96]. **E)** Diagram illustrates the SGM preparation process and the mechanism of SGM treatment of colitis and intestinal colonization [97]. **F)** Preparation of HPN by self-assembly of HA-PPS molecule, encapsulation of EcN with the NE layer, and conjugation of HPN to the surface of EcN [105]. **G)** Schematic illustration for the preparation of coated EcN by DA assisted co-deposition with functional small molecules and polymers. Representative TEM images of native and PDA-coated EcN. Scale bar: 400 nm [99]. **H)** Preparation and characterization of EcN-Apt@HG [108]. **I)** Synthetic procedure of HABN and BL@HABN [110].

MPN-encapsulated probiotics show promise for IBD treatment by utilizing multifunctional coatings constructed through coordination due to its various properties, such as selective permeability, enhanced mechanical and thermal stability, and stimuli responsiveness [81,82]. The multifunctional strength of hybrid probiotic coatings stems from the synergistic interplay of complementary components, providing enhanced protective and therapeutic benefits. Polyphenol molecules can rapidly assemble iron ions in aqueous solutions through chelation coordination to form MPN. Due to the strong adhesive properties of phenolic molecules on various solid surfaces, adding polyphenol and iron ions to the solution using different solids as templates can create MPN surface coatings on the solid surfaces. The adjacent hydroxyl groups of polyphenols serve as chelating sites for metal ions, and the numerous gallol groups on polyphenol enable efficient coordination-driven cross-linking, forming three-dimensional MPNs. The multiple interaction forces formed by catechol and pyrogallol in the polyphenol molecular structure serve as the chemical basis for regulating mass transfer across different phases and at micro-nano interfaces [83]. Polyphenols form various intermolecular interactions, including hydrogen bonding, π-π stacking, and electrostatic interactions, which allow them to functionalize surfaces with diverse chemical properties. This enhances interactions between the functionalized surfaces and other substances, leading to strong adsorption capacity and selectivity [84]. Polymers such as mucin or β-glucan can interpenetrate the MPN, creating a denser, more robust network that resists enzymatic degradation and mechanical shear significantly better than an MPN alone. A multi-layer modification system composed of mucin/polyoxamers188/β-glucan not only enhances storage stability but also endows probiotics with mucus penetration and free radical scavenging capabilities, extending intestinal colonization time to over 48 h [85]. In this system, Fe^3+^ was anchored to the surface of EcN through electrostatic adsorption. Subsequently, a TA/Fe^3+^ network layer was formed via the cross-linking of galloyl groups and Fe^3+^ ions. Finally, a layer of mGN was deposited onto the TA/Fe^3+^ layer due to the attraction of numerous hydrogen bonds between the TA/Fe^3+^ network and mGN, forming a modified prebiotic-based shield (Fig. 6A). The entire preparation process took approximately 15 min. Live probiotics are increasingly being recognized as potential therapeutic agents for intestinal diseases, owing to their capacity to inhibit pathogen colonization and actively modulate the composition of the gut microbiota [86]. Nevertheless, these therapeutic interventions are often administered concurrently with antibiotic treatments that target pathogenic microorganisms. In such clinical scenarios, the efficacy of probiotics is frequently compromised due to their inherent sensitivity to the antimicrobial effects of antibiotics. TA/Fe^3+^ composite layers can resist gastric acid erosion while addressing the challenge of co-administration of probiotics and antibiotics by adsorbing antibiotics.

To enhance the retention time of probiotics in the intestine, TA is commonly used in the modification process [87]. TA, a secondary metabolite produced by microorganisms and plants, has a molecular weight of approximately 500–3000 Da. The catechol groups in TA can form hydrogen bonds, covalent bonds, and/or π-π interactions with various substrates, giving TA strong adhesive properties [88]. Hu et al. [89] prepared nanostructured pBDT-TA through the self-aggregation of aromatic dithiols (BDT) and TA, which exhibited excellent antioxidant and anti-inflammatory properties *in vitro*. The pBDT-TA was LbL coated onto the surface of EcN with sodium AG to construct the EcN@SA-pBDT-TA delivery system. The modified probiotics demonstrated resistance to oxidative and inflammatory stress, enhancing colon-targeting and retention capabilities in IBD model mice. Pan et al. [87] developed a biocompatible supramolecular coating composed of TA and iron ions, referred to as a nanoshield, which can protect probiotics from antibiotic interference. This nanoshield strategy enhances the efficacy of therapeutic probiotics in the GIT of patients undergoing antibiotic treatment, mitigating the negative impact of antibiotics on the gut [90].

Mucin, an acid-resistant glycoprotein, is present in the mucus of the GIT. Composed of mucopolysaccharides, it interacts with the intestinal mucus layer through hydrogen bonding, disulfide bonding, and hydrophobic forces [91]. Given that an intestinal mucus layer is a suitable place for the colonization and growth of gut microbiota, multiple TA monomers can react with Ca^2+^ to form a nanofilm that adheres to mucin. Yang et al. [91] developed a strategy for a super probiotic (EcN@TA-Ca^2+^@Mucin) coated with TA and mucin using LbL technology. The research demonstrated that mucin enhances the resistance of probiotic to the harsh GIT environment and improves their adhesiveness to the intestines through interaction with mucus, which promotes colonization and growth within the mucus layer without removing the coating. This hybrid coating employs a robust TA-Ca^2+^ network as its adhesive foundation, topped with a critical layer of mucin which not only confers superior acid resistance but also acts as a biomimetic camouflage, enabling the probiotic to seamlessly integrate with the mucus layer and dramatically enhance colonization without requiring coating removal(Fig. 6B).

#### 3.2.2 Covalent conjugation

Covalent conjugation strategies for bacteria utilize abundant reactive groups in chemical reactions to achieve stable binding between functional molecules, macromolecules, nanoparticles and the bacterial surface [92]. Bacterial cell walls contain various surface proteins with distinct functional groups. The most common are amino, carboxyl, and thiol groups. These groups are easily manipulated, highly efficient, and function under mild conditions, making them ideal sites for chemical modification of the bacterial surface [93]. Thiol-disulfide exchange without catalysts is achieved through nucleophilic substitution by thiol anions, with its rate strictly regulated by pH and the pKa of the thiols. Although the kinetics are slow, its bioorthogonality, redox responsiveness, and dynamic reversibility make it indispensable in the fields of biomaterials and drug delivery. Luo et al. [80] developed a method for covalent bacterial localization via in-situ chemical reactions. Using a one-step imidoester reaction, this approach converts surface amino groups into thiols on bacteria. The technique is applicable to various bacterial strains, with adjustable thiol levels via feed ratio modulation. The modified bacteria spontaneously bind to the mucosal layer through catalyst-free thiol-disulfide exchange between mucin-associated disulfides and bacterial surface thiols, exhibiting attachment dependent on thiolation levels (Fig. 6C).

Bacterial adaptability to environmental changes involves various cellular mechanisms, including redox enzyme cascades. These reactions have been utilized for the synthesis of artificial polymers. For instance, catechol-containing phenolic compounds can be surface-modified through oxidative covalent cross-linking. Tea polyphenols, such as gallic acid (GA), epigallocatechin (EGC), and epigallocatechin gallate (EGCG), are biocompatible, biodegradable, and among the most potent dietary antioxidants due to their strong reducing properties [94,95]. Their inherent phenolic structure indicates significant potential for various biomedical applications, including probiotic delivery. A novel self-assembling coating composed of MPNs, which consists of non-covalent coordination complexes formed by Fe^3+^ and natural polyphenols absorbed on bacterial surfaces through non-covalent interactions. Gao et al. [96] evaluated three tea polyphenols- GA, EGC, and EGCG-combined with ferric ions to coat *Lactobacillus rhamnosus* LGG (MPN@L), comparing them against TA-based MPN@L for IBD treatment efficacy. All MPN@L formulations showed enhanced adhesion and retention in *ex vivo* and *in vivo* models compared to uncoated probiotics. All MPN@L systems alleviated UC by reducing myeloperoxidase levels, modulating cytokines, and improving gut microbiota composition. EGC@L specifically enriched beneficial genera (*Lactobacillus, Adlercreutzia, Oscillospira*) while suppressing pro-inflammatory taxa. This work highlights MPN-based probiotic modification as a viable strategy for gastrointestinal therapeutics, broadening the scope of probiotic applications in IBD management (Fig. 6D).

#### 3.2.3 Self-assemble

Recently, Yang et al. [97] developed a super gut microorganism with a self-assembling TA and poloxamer 188 coating for improved intestinal colonization. This coating resists physiological challenges, modulates pathological conditions by scavenging inflammation-induced ROS and depriving pathogenic bacteria of iron, and enhances probiotic survival. Moreover, it promotes strong adhesion to intestinal mucosa, particularly improving probiotic colonization in diseased states (Fig. 6E).

#### 3.2.4 Oxidative polymerization

Inspired by the remarkable adhesion and biocompatibility of mussel adhesive proteins, a range of catechol-based molecules such as dopamine (DA) and TA have been used to modify various biomaterial surfaces. Both *in vivo* and *in vitro* studies have demonstrated that DA-based coatings can improve the resilience of bacteria to gastrointestinal stress without impacting their viability [98,99]. Emerging findings indicate that interaction with the intestinal mucosal barrier serves as the primary pathway for communication between symbiotic bacteria and the host [100,101]. Due to the presence of intrinsic amino and hydroxyl groups on cell membranes, the oxidative self-polymerization of DA is widely used to introduce multifunctional materials onto cell surfaces. PDA exhibiting strong adhesive properties [102], can flexibly deposit various functional small molecules and macromolecules onto bacterial surfaces through hydrogen bonding, π-π stacking, Michael addition, and Schiff base reactions [103]. Li et al. [98] utilized PDA to create biocompatible coatings on microbial surfaces through hydrogen or covalent bonding, increasing intestinal bioavailability by 30 times. The derivative, polydopamine nanoparticular immunosuppressant (PDNI), not only protects probiotics but also regulates the stimulate regulatory T (Treg) cells/T helper 17 (Th17) balance, significantly alleviating UC. Norepinephrine (NE), both a hormone and neurotransmitter, can form a polyNE membrane on the surface of probiotics via self-oxidation [104]. Liu et al. [105] synthesized HA-polypropylenesulfone (HPN), which self-assembles into nanoparticles capable of efficiently removing ROS. These nanoparticles covalently bind with NE-encapsulated EcN probiotics forming to protect probiotics (NE-EcN), where the NE layer enhances the tolerance of EcN to the GIT environment and mucosal adhesion, extending its intestinal retention time. HPN-NE-EcN exhibited anti-inflammatory and ROS scavenging effects, effectively preventing and treating UC while enhancing intestinal microbiota diversity and beneficial bacteria abundance (Fig. 6F). Pan et al. [99] demonstrated that by introducing a small amount of CS during the polymerization process of DA, a co-deposited coating of PDA and CS can form on the surface of probiotics. This enables effective accumulation of probiotics in the colon, essentially providing them with a protective layer equipped with navigation capabilities. This coating enhances their resistance to gastric acid and bile erosion while improving their targeted delivery to disease sites, thereby contributing to improved efficacy in the treatment of UC(Fig. 6G).

The functional significance of repairing the intestinal barrier lies in its capacity to restore systemic immune homeostasis, improve gut physiological function, and potentially impact extra-intestinal organ systems, thereby positioning it as a fundamental therapeutic target. Chen et al. [106] propose leveraging bacterial PEGylation to enhance intestinal mucosal barrier function. By utilizing DA self-polymerization, amine-terminated poly(ethylene glycol) (m-PEG-NH_2_) conjugates with PDA, forming a coating on bacterial surfaces under cyto-compatible conditions. This PEGylation creates a hydrophilic, net-neutral surface that improves bacterial mobility and mucus penetration. Enhanced penetration promotes bacterial retention in the mucus layer, which blocks pathogenic invasion to preserve gut microbiota balance while stimulating mucus secretion and tight junction proteins such as occludin and zonula occludens 1 (ZO-1) expression in the lower GIT. Besides traditional polyelectrolytes, hydrophobically modified hydrogen silicate nanosheets acquire positive charges and form protective coatings through electrostatic interactions, which stabilizes in gastric acid, rapidly degrades in the intestine to release hydrogen gas, and can be used for probiotic surface modification [47].

#### 3.2.5 Amidation

Interleukin-6 (IL-6) is a vital pro-inflammatory cytokine that accelerates the progression of UC by promoting chronic inflammation and tissue damage. Elevated IL-6 levels are consistently found in the mucosa of UC patients. IL-6 aptamers specifically identify and bind to IL-6 molecules, facilitating the targeted delivery of therapeutic agents to inflamed regions [107]. Chen et al. [108] conjugated IL-6 aptamers onto the surface of EcN through a simple cell-compatible amidation method, forming aptamer-modified probiotics (EcN-Apt). Subsequently, the aptamer-modified EcN was encapsulated in a hydrogel containing oxidized chondroitin sulfate and polyamidoamine dendrimers, ultimately developing a novel hydrogel for oral probiotic delivery. The IL-6 aptamers on the probiotic surface specifically bind to high levels of IL-6 in inflamed intestinal tissues, promoting significant accumulation of probiotics in areas of intestinal inflammation (Fig. 6H). The capacity of bacteria to adhere to intestinal mucus for extended periods is essential for their physiological functions. *Bifidobacteria* naturally adhere to the intestinal mucus layer through surface adhesion proteins, such as pili, moonlighting proteins, extracellular vesicles, and other surface-anchored proteins [109]. Wang et al. [110] developed a mucosal adhesion-mediated therapeutic strategy using probiotics, employing ROS scavengers to modulate gut inflammation and microbiota for biological adhesion. *Bifidobacterium longum* (BL), a mucosal-adherent oral probiotic, was loaded with HA-bilirubin nanoparticles (HABN) to form BL@HABN. BL@HABN adheres to epithelial cells and exhibits prolonged retention time, mediating sustained release of pre-loaded HABN nanoparticles (Fig. 6I).

#### 3.2.6 Boronic acids and cis-diols click reaction

Click Chemistry is extensively used in the pharmaceutical and biotechnology sectors for bioconjugation, biomarking, and materials science, due to its mild conditions and high selectivity. The reaction is inherently efficient, modular, rapid, and environmentally friendly [111]. Click reactions between boronic acids and cis-diols are rapid (often seconds) and occur under mild conditions (neutral pH, aqueous solvent, room temperature, mild pressure), minimizing disruption to cell surface proteins and physiological processes [112].

**Fig. 7:**
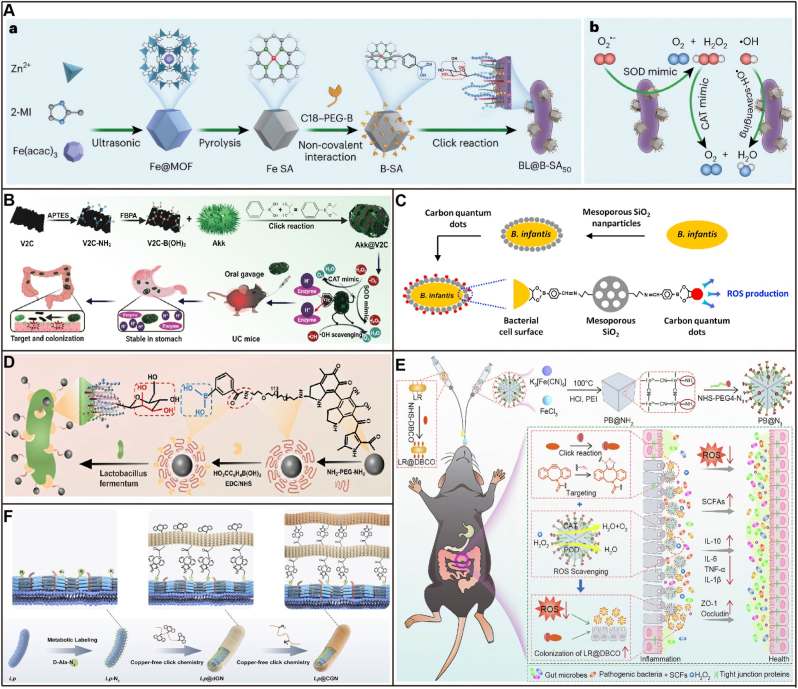
Surface chemical modification of probiotic. A) a) Preparation of artificial-enzyme-armed probiotics. BL@B-SA_50_. b) BL@B-SA_50_ can mimic SOD and CAT antioxidant enzymes as well as function as antioxidant molecules to scavenge multiple ROS to regulate the fate of cells and microbes [113]. **B)** Schematic illustration of MXenzyme armed probiotics and the mechanisms for inflammation-targeted delivery and synergistically therapy for UC [114]. **C)***B. infantis* ATCC 15697 encapsulation in a boron hydroxyl-modified, mesoporous silica nanoparticle layer with attached bacterially derived CQDs [98]. **D)** Schematic representation of DSS-induced UC mice treated with Lf@MPB [115]. **E)** Schematic diagram illustrates the preparation of PB@N and its active mechanism in enhancing targeted colonization of LR@DBCO for treating IBD [119]. **F)** Demonstration of a synthetic procedure for Lp@CGN by bioorthogonal LbL coating strategy [129].

In the context of IBD, strict anaerobic probiotics are especially vulnerable to oxidative damage from ROS due to the lack of antioxidant enzymes. To address this problem, Mao et al. [113] modified artificial enzymes with ROS capabilities onto *Lactobacillus longum* probiotics to construct an anti-inflammatory engineered probiotic (BL@B-SA_50_) for regulating intestinal inflammation. This engineered probiotic consists of three parts: artificial enzymes, probiotics, and a linker. The artificial enzyme selected is iron single-atom catalyst Fe SA, which has stronger antioxidant capacity than traditional nanoenzymes, enabling rapid clearance of ROS and protection of intestinal cells and probiotics. The probiotic chosen is *Bifidobacterium longum* (BL), which not only aids in IBD repair but also possesses gut colonization ability, extending the targeted retention time of the artificial enzyme in the intestine, thus continuously clearing excessive ROS and inflammatory factors (Fig. 7A). The specific binding of boric acid with cis-1,2-diol essentially involves the formation of a five-membered cyclic borate ester through stereospecific matching. By utilizing the ortho-amino effect and molecular engineering strategies, highly sensitive sugar recognition can be achieved under physiological conditions, providing an indispensable chemical tool for enzyme-free sensing, targeted delivery, and intelligent materials. In addition, Peng et al. [114] designed a multienzyme mimicking vanadium carbide (V2C) MXenzyme armored colon-targeting *Akkermansia muciniphila* (Akk) probiotic to synchronously alleviate inflammation and regulate microbiota. V2C is induced on boron hydroxyl groups and coated onto the probiotic via boronic acid vicinal-diol-based click reactions and called Akk@V2C. V2C surface modification shields Akk from gastrointestinal harm. Akk and electrostatic adsorption enable V2C to target lesions. V2C clears lesion ROS, protecting Akk from oxidative stress, promoting its colonization, thus enhancing the gut microbiota and aiding in the treatment of UC. Akk@V2C exhibits notable curative effects by alleviating inflammation, reprogramming macrophage polarization, and regulating microbiota homeostasis, thus promoting SCFAs production and restoring intestinal barriers by upregulate the expression of occludin and ZO-1 (Fig. 7B). The restoration of the intestinal barrier and the regulation of tight junction proteins, as demonstrated in our study, confer functional benefits that extend well beyond the modulation of gut microbiota composition. While the stabilization of a healthy microbial community is an important outcome, the direct reinforcement of the physical barrier is paramount for systemic homeostasis. Also, pathogen-derived CQDs can inherit diverse chemical groups and functions from their source bacteria, resulting in the production of ROS with a wider range of activities. Wei et al. [48] utilized CQDs to modify probiotics, enhance survival rate and antagonistic effects against pathogens, providing a new strategy for single-cell surface modification of probiotics (Fig. 7C). Additionally, Liu et al. [115] developed a targeted oral formulation for functionalized probiotic Lf@MPB, centered on *Lactobacillus fermentum* (Lf), where melanin nanoparticles (MNP) were modified onto its surface via a tricarboxy phenylboronic acid click reaction, aiming for synergistic treatment of UC. Results indicated that Lf@MPB has strong ROS scavenging ability and significantly enhances the viability of Lf probiotics in simulated gastrointestinal fluids. *In vivo* fluorescence imaging showed that Lf@MPB accumulates extensively at sites of intestinal inflammation induced by dextran sulfate sodium in UC mice (Fig. 7D). Chen et al. [116] found that zinc (Zn) and indole-3-carbinol (I3C), derived from diet, exhibit a unique synergistic effect in repairing the intestinal epithelial barrier by upregulate the expression of occludin and ZO-1. A platform includes Zn, I3C, and 2-methylimidazole (2-MIM) assembled in a one-pot method for the sustained and simultaneous release of both compounds was developed. The ZI was then conjugated to the surface of EcN using a C18-PEG-phenylboronic acid linker. ZI@EcN demonstrated robust preventive and therapeutic effects with high safety.

#### 3.2.7 Azide and dibenzocyclooctene click reaction

Click chemistry, particularly bioorthogonal copper-free variants, is a powerful tool for creating stable, functional linkages on probiotic surfaces under physiological conditions. The spatiotemporal guidance relies on the decoration with azide (N) groups and dibenzocyclooctene (DBCO) groups. This process is known as copper-catalyzed azide-alkyne cycloaddition or click chemistry [117]. Song et al. [118] developed a click chemistry-mediated bacterial delivery strategy to enhance probiotic colonization by modulating bacterial adhesion between probiotics and gut bacteria. Azide-modified alanine integrates azide groups into the bacterial cell walls of gut microbiota via amino acid metabolic engineering. Even in complex physiological environments, DBCO-modified bacteria can produce significant bacterial adhesion with azide-modified bacteria through bioorthogonal conjugation. This strategy highlights metabolically modified gut bacteria as reactive sites for click chemistry reactions with DBCO-modified probiotics, thereby enhancing probiotic delivery and colonization. Dong et al. [119] developed an azido (N_3_)-modified prussian blue nanozyme (PB@N_3_) that enhances targeted probiotic colonization via spatio-temporal guidance to alleviate intestinal inflammation. The PB@N_3_ selectively targets inflamed intestinal regions while scavenging ROS. Biorthogonal click chemistry then enables precise spatio-temporal colonization of DBCO-modified *Lactobacillus reuteri* DSM 17938 (Fig. 7E). In addition, Peng et al. [120] introduced a practical approach utilizing biorthogonally functionalized probiotics to alleviate IBD by modulating inflammatory cytokines, enhancing the intestinal barrier by upregulate the expression of occludin and ZO-1, and adjusting the gut microbiota. They modified EcN with azide ligands via N-hydroxysuccinimide ester (azido-PEG4-NHS ester) coupling with the amine groups present on the bacterial cell wall. High molecular weight HA was then conjugated to DBCO and attached to the EcN surface using azide-alkyne click chemistry. This HA-functionalized EcN (EcN-HA) was subsequently encapsulated with Eudragit L100-55 through calcium ion-assisted electrostatic interactions (EcN-HA@L). This encapsulation protects the probiotics from deactivation in the acidic stomach environment and disintegrates upon reaching the neutral pH of the intestines.

β-Glucan (GN) could enhance intestinal health by improving the microenvironment, balancing flora, and repairing the gut barrier. Also, resistant to degradation in the upper GIT and biodegradable bioactivity in the intestines make GN an ideal coating material for oral probiotic delivery systems [85]. The copper-catalyzed azide-alkyne cycloaddition (CuAAC) reaction was reported and has since become a landmark reaction in click chemistry [121,122]. Its mild reaction conditions, which are insensitive to water and oxygen, along with its biorthogonality-due to the rarity of azides and alkynes in the body-have significantly advanced its use in biomedicine, such as in site-specific protein modification [123] and functional modification of cell surfaces [124]. However, free Cu(I) can undergo redox reactions in the body, producing harmful free radicals and exhibiting notable cytotoxicity, which severely restricts the application of CuAAC in active biological systems [125,126]. To address this issue, the copper-free click chemistry strategy emerged, driven by the ring strain caused by the geometric deformation of the alkyne. This strategy, known as the strain-promoted azide-alkyne cycloaddition (SPAAC) reaction, retains the specificity of the azide and alkyne groups while offering improved biocompatibility [127]. Cyclooctyne reacts with azides under mild temperature and pressure conditions to form a mixture of triazole positional isomers, without requiring metal catalysts and showing no significant cytotoxicity [128]. Ji et al. [129] devised a copper-free click chemistry strategy by coating GN prebiotic onto the surface of *Lactobacillus plantarum* (Lp) probiotic at the single-cell level (Lp@CGN) based on bioorthogonal chemistry in a LbL manner. This achieved to form a firm, dense, and multifunctional GN-based armor with advances of superior protective properties, colon-targeted degradation, and prebiotic benefits. Under the protection of the prebiotic-based armor, Lp@CGN exhibited a notable 276-fold increase in the survival rate compared to naive Lp after exposure to whole GI conditions. Upon reaching the colon, the “armor” was metabolized into SCFAs by gut microbiota, facilitating the timely release of Lp within colon, thereby achieving a synergistic treatment effect due to sustained SCFAs generation and Lp liberation (Fig. 7F). Peng et al. [130] developed a practical strategy for creating antioxidant and immunomodulator nanoengineered probiotics for IBD synergistic therapy. They functionalized EcN with bilirubin (BR), a natural antioxidant, via amide condensation, and conjugated it with HA using bio-orthogonal coupling (EcN-BR/HA). This dual modification protects probiotics from gastric acid and digestive enzymes. Upon reaching the neutral pH of the intestinal tract, the HA coating swells, allowing the probiotics to regain their activity due to the ionization of carboxyl groups.

### 3.3 Biological modifications

Microorganisms possess metabolic capabilities such as reducing metal ions, producing biofilms, and binding specific surface receptors, which can be used for the modification of single-cell and probiotic cells [51]. The method of encapsulating probiotics using microbial metabolic activity is referred to as bio-modification. Among common bio-modification approaches, biofilm and spore approaches are primarily defensive, with spores providing superior passive protection for viability during transit and biofilms excelling at promoting active gut adhesion. In contrast, genetic engineering is transformative and offensive, fundamentally enhancing probiotic functionality to enable precise colonization and turn it into a self-sustaining, localized therapeutic production unit for superior long-term durability. The choice hinges on therapeutic goals: genetic engineering offers the most potent and durable effect, biofilms provide a readily translatable path for improved persistence, and spores are ideal for ensuring survival through harsh storage and GIT.

#### 3.3.1 Spores

**Fig. 8:**
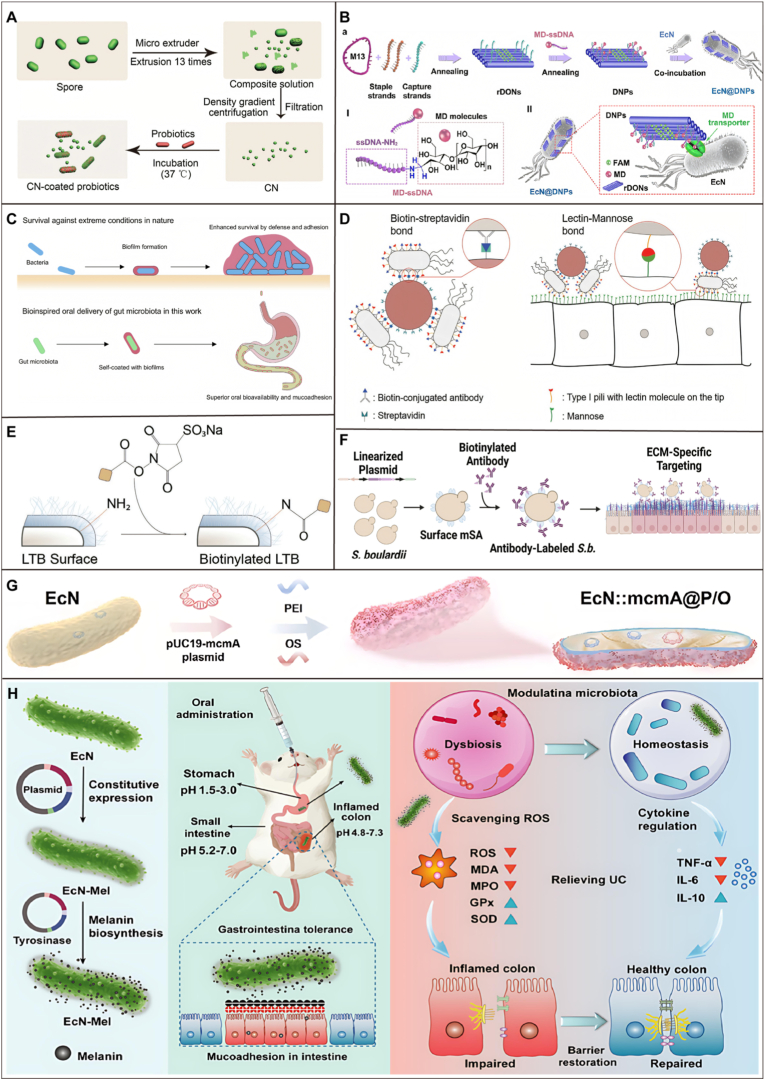
Surface biological modification of probiotic. A) Schematic of process of CN preparation from probiotics spores [132]. **B)** Design, synthesis, and characterization of EcN@DNPs. (a) Schematic representation depicting the design, preparation, and synthetic pathways of rDONs, DNPs, and EcN@DNPs. Inset I illustrates the covalent linkage between MD and ssDNA via a Schiff-base reaction to form DNPs. Inset II highlights the interaction between MD in DNPs and the MD transporter on the exterior of EcN, leading to the formation of the EcN@DNPs system [137]. **C)** Schematic illustration of biofilm formation of bacteria in nature to enhance survival by defense and adhesion under extreme conditions and bioinspired oral delivery of gut microbiota with superior oral bioavailability and mucoadhesion by self-coating with biofilms [141]. **D)** Schematic of bacteria-bots consisting of synthetic microparticles with attached bacterial cells using the biotin-streptavidin bond and attaching to mannose-expressing disease site cells through lectin-mannose bond. Lectin molecule is located on tip of bacterial type I pili [148]. **E)** Schematic of N-hydroxysulfosuccinimide ester chemistry for bioconjugation of biotin to primary amines on the bacteria surface [149]. **F)** Schematic of engineering steps to enable the binding of *S.b.* to extracellular matrix proteins [154]. **G)** Schematic of the preparation of EcNmcmA@P/O [157]. **H)** Graphic Illustration of Oral EcN-Mel Treatment Methods for Colitis [158].

Microbial spores exhibit high resistance to pressure and high temperatures due to their low water content and germinate and colonize in the gut under specific nutritional triggers [131]. Song et al. [132] transformed spore coats into multifunctional nanoparticles via mechanical force extrusion, significantly enhancing probiotic functions such as regulating microbiota, maintaining barriers by upregulate the expression of occludin and ZO-1. This technology is adaptable to various strains, such as loading chemotherapeutic drugs into spores via deoxycholic acid modification and utilizing bile acids to enhance nanoparticle internalization for targeted drug release in the intestine (Fig. 8A).

#### 3.3.2 DNA origami

DNA origamis, created by folding long viral single-stranded DNA (ssDNA) with multiple short ssDNA, have emerged as advanced agents to neutralize oxidative stress [133,134]. Additionally, DNA origamis have been widely used in drug delivery, therapy, and diagnosis due to their addressability, programmability, and biocompatibility [135,136]. However, the effectiveness of DNA origamis in treating IBD is hindered by their poor retention in the colon. Combining DNA origamis with probiotics could potentially enhance probiotic retention in the colon, but the unique plasma membrane and cell wall of bacteria create electrostatic charges or barriers that complicate the modification of DNA origami to the bacterial surface. He et al. [137] constructed bacteria-specific DNA nanostamps for the modification of engineered EcN triggered by ultrasound. DNPs consist of rectangular DNA origami nanosheets capable of clearing ROS and maltodextrin molecule ligands targeting bacteria, which can selectively attach to bacterial surfaces. Additionally, the bioavailability of probiotics in the GIT was enhanced through the self-assembly strategy of CS and sodium alginate. In a UC mouse model, this system significantly repaired the intestinal barrier by upregulate the expression of occludin and ZO-1 and effectively alleviating inflammation (Fig. 8B).

#### 3.3.3 Biofilms

Biofilms are communities of surface-adhered microbes embedded in a self-produced extracellular matrix containing proteins, eDNA, polysaccharides, and lipids [138,139]. Introduced gut bacteria unable to form biofilms are rapidly cleared, even in abundance, explaining the limited efficacy of many probiotic strains [140]. Therefore, biofilms could be used to enhance the survival of bacterial cells under extreme conditions. Wang et al. [141] encapsulated *Bacillus subtilis* with biofilms, rendering it highly resistant to gastrointestinal fluids and ampicillin, a characteristic attributed to the hydrophobic nature of biofilms that impedes toxin diffusion. Genetically engineering probiotics to produce specific biofilms is a versatile strategy for oral gut microbiota delivery. The ability to survive in complex environments suggests these biofilm-coated probiotics could also be used in respiratory and reproductive tract infections [142] (Fig. 8C). Given that self-coating with biofilms had been reported with the capacity of improving bacterial resistance and adhesion in GIT [141,143], Guo et al. [144] proposed a strategy for co-delivering *Lactobacillus casei* (Lac) and highly active selenium. A nutrient deficiency culture induced a bacterial pericellular film on *Lactobacillus casei* (Lac), and the spatial confinement of its internal polysaccharide network was utilized to embed ultra-small selenium points (Se-fLac) on the bacterial surface. After oral administration, Se-fLac effectively enhanced the tolerance of Lac to gastric acid and intestinal adhesion. At the site of UC, the selenium efficiently removed ROS, while Lac regulated imbalanced gut microbiota, restoring intestinal redox homeostasis and microbiome balance, contributed to a synergistic therapeutic effect against UC.

The use of cell-mediated catechol chemistry is an emerging strategy that harnesses the metabolic processes of living cells to create biomimetic materials with advanced functionalities and enhanced biocompatibility. A cell-mediated catalytic process for forming protective nano-shells on individual probiotic cells is demonstrated. Franco Centurion et al. [145] employed Lac biocatalysis to oxidize catechol compounds such as DA and caffeic acid, forming protective coatings on probiotics. This process, initiated by manganese within bacterial cells, significantly boosts the oxidation rate of phenolic compounds. This improvement enhances the probiotics survival in gastric acid by about 1.4 times without additional functionalization. It also improves adhesion and antioxidant activity, setting the stage for IBD treatment.

#### 3.3.4 Receptor-ligand binding

Antigen-antibody interactions and streptavidin-biotin conjugation are widely used, specific, and reversible receptor-ligand methods for the surface modification of live bacterial drugs [146]. Biotin, known for its safety and effectiveness as a synthetic adhesin Biotin and streptavidin interact specifically to form a synthetic adhesin, which is widely used in biological coupling reactions [147]. Mostaghaci et al. [148] constructed drug-loaded microbe-*E. coli* complexes using mannose-specific binding to prolong drug retention time (Fig. 8D). Vargason et al. [149] enhanced pathogen clearance efficiency by fixing biotin on the surfaces of *Lactobacillus* species through esteramine chemistry (Fig. 8E).

Adhesins are crucial for establishing spatial niches within the GIT, supporting microbial survival and proliferation [150]. The expression of specific membrane ligands or receptors through synthetic bioengineering or the addition of adhesive functional groups via physicochemical methods has been widely studied to enhance bacterial interactions with host extracellular matrix proteins like fibrinogen and collagen [[151], [152], [153]]. Heavey et al. [154] genetically engineered a targeted probiotic platform where *Saccharomyces boulardii* expresses monomeric streptavidin on its cell surface, functioning as a connector for extracellular matrix (ECM) -specific biotinylated binding to abundant ECM proteins present in GIT inflammatory lesions, utilizing tunable antibody surface display. The results showed that engineered probiotics had 350 times higher binding capability with relevant ECM proteins compared to non-targeted strains and maintained at least 15 times higher binding capability after 48 h in a simulated gastrointestinal environment (Fig. 8F).

#### 3.3.5 Genetic engineering

With the rapid advancement of gene sequencing and editing technologies, genetic engineering has become widely used to analyze and modify biological genomes. Gene editing allows for the integration of foreign genes and precise modification of specific endogenous genes, enabling the engineering of bacteria to alter their features and functionalities [155]. Through genetic engineering, target cells can be programmed to express or secrete a diverse range of exogenous functional molecules, such as antigens, antibodies, and enzymes, thereby enhancing critical bacterial capabilities including targeting, adhesion, and metabolite synthesis. [156]. Decorating the bacterial surface using gene-editing technologies is a well-established strategy, often achieved through the incorporation of exogenous target genes contained in recombinant plasmids or integrated into the genome. Jiang et al. [157] developed a strategy leverages bacterial programmability and gene editing to create bactericidal agents that dynamically adjust the intestinal microecology and use controlled chemical modifications to boost bacterial resistance. Using EcN as a model, they developed a polyelectrolyte composite coating in a single step through electrostatic and hydrogen bonding interactions. This genetically engineered strain (EcNmcmA) acts as a live cell factory programmed to synthesize MccM in situ. The MccM utilizes a "Trojan horse" mechanism, mimicking iron-chelating siderophores to be internalized by competing bacteria via their TonB-dependent outer membrane receptors, ultimately disrupting their cell membranes and causing death. To ensure this engineered probiotic survives the harsh gastrointestinal tract to deliver its payload, a protective polyelectrolyte composite coating was applied in a single step through electrostatic and hydrogen bonding interactions. This coating, composed of oxidized starch and polyethylenimine (PEI), leverages a physical barrier effect and a protophilic effect, drastically enhancing bacterial survival (40-fold in the stomach and 74-fold in the small intestine). This dual strategy leverages synthetic biology to program bacteria for the in-situ synthesis of therapeutic agents and modulates the gut microbiome, while employing controlled surface modification to bolster probitic resilience within the harsh gastrointestinal tract. (Fig. 8G). Liu et al. [158] obtained a biosynthetic melanin-producing engineered strain (EcN-Mel) from genetically modified ECN expressing tyrosinase genes and evaluated the feasibility and efficacy of oral administration of EcN-Mel in a UC mouse model. The hydroxyl groups on the melanin surface can form hydrogen bonds with polar functional groups in proteins and polysaccharides at inflammation sites (Fig. 8H). The hydroxyl groups on the melanin surface can form hydrogen bonds with polar functional groups in proteins and polysaccharides at inflammation sites, strengthening the bond between melanin nanoparticles and these molecules. This interaction further stabilizes the binding between melanin nanoparticles and biomolecules in the inflammatory microenvironment, leading to increased accumulation of melanin at the site of inflammation. Consequently, this enhanced targeting strategy not only improves the therapeutic effectiveness of melanin nanoparticles but also minimizes potential systemic side effects [159]. This strengthens the bond between melanin nanoparticles and these molecules, further stabilizing their interaction in the inflammatory microenvironment. As a result, there is an increased accumulation of melanin at the site of inflammation. The results indicate that EcN-Mel produces and secretes melanin, exhibiting targeted intestinal adhesion, radical scavenging ability, and gastrointestinal stability.

## 4 Surface modification technology applications for IBD therapy

Briefly, Physical interactions primarily depend on non-covalent forces, such as hydrogen bonding, van der Waals forces, and electrostatic interactions. Chemical interactions, by contrast, are characterized by the formation of covalent bonds or coordination bonds, which involve the sharing or transfer of electrons between atoms. Biological interactions, on the other hand, leverage the inherent metabolic processes and structural properties of cells, enabling functions such as enzyme catalysis, signal transduction, and molecular recognition through the precise arrangement of biomolecules like proteins, nucleic acids, and carbohydrates. Surface decoration of individual probiotis cells through physical, chemical, and bioengineering approaches offers promising pathways for novel biotherapeutics. Physical methods utilize non-covalent interactions for mild, reversible modification suitable for drug delivery, though their instability and lack of specificity limit long-term applications. Chemical approaches employ covalent bonding to functionalize cell surfaces, enabling stable and high-load modifications, yet require careful optimization to preserve cell viability. Bioengineering techniques, including genetic editing, allow precise enhancement of probiotic functions such as GI tract tolerance and targeted action, though potential biosafety issues like antibiotic resistance and uncontrolled colonization remain concerns.

Compared to physical methods, chemical and bioengineering strategies show stronger clinical potential. Chemical modification with clinically approved materials can reduce immunogenicity and improve targeting, while genetic engineering enables persistent function with higher biocompatibility. Future efforts should focus on developing mild chemical techniques, biocompatible materials, and standardized self-assembly strategies to jointly advance modification stability, functionality, and biosafety.

The choice of probiotic strain is critical and should be guided by the desired therapeutic outcome. For example, EcN is ideal for active immunomodulation, LGG for robust delivery and combination therapy, and BL for sustained mucosal barrier protection and repair. The surface modification strategies are then tailored to overcome the specific limitations and amplify the innate strengths of each strain. We have incorporated a discussion of these important inter-strain differences in the revised manuscript to provide a more nuanced perspective.

### 4.1 Clinical progress

Single-cell surface modification technology for probiotics holds transformative potential in biotechnology by enhancing their therapeutic efficacy, survivability, and targeted delivery [10]. These protective systems operate through multiple mechanisms. They form a physical barrier against adverse conditions such as high temperature, humidity, oxygen, and gastric acid, thereby drastically reducing probiotic inactivation during transit. Upon reaching the intestinal tract, these smart carriers can release their probiotic payload in response to specific physiological stimuli like pH or enzymes. Furthermore, the functional groups on the carrier materials can engage in robust interactions with intestinal mucins, significantly enhancing the adhesion and colonization potential of the probiotics, which is a critical determinant for their sustained beneficial effects.

The core of this progress lies in the development of advanced encapsulation strategies. Delivery carriers, including those based on LbL assembly and biomimetic cell membranes, have demonstrated substantial potential in shielding probiotics from the rigors of processing, storage, and the harsh gastrointestinal environment [40,72]. The probiotic regulations of Asia-Pacific, United States, and Europe have clearly stipulated that active probiotic health foods must have a live bacterial count of no less than 10^6^ CFU within their shelf life [162], indicating that the live bacterial rate is crucial for probiotic products [163]. The implementation of single-cell coating technologies is a pivotal strategy to meet this stringent requirement, ensuring that enough live bacteria reach the intended site of action to exert their therapeutic functions. By exploiting the probiotic's innate capacity for gut colonization and enhancing it with a protective, stimulus-responsive coating, these integrated systems ensure the coordinated release of both viable bacteria and their therapeutic drug cargo precisely at the site of intestinal inflammation.∗∗The synergy in these advanced formulations arises from a coordinated division of labor, where the modified probiotic acts as a smart, targeted delivery vehicle and microenvironment modulator, while the co-delivered drug provides precise pharmacological action, together creating a multi-pronged, self-reinforcing therapeutic system that is more effective than the sum of its parts.

### 4.2 Clinical translation, regulatory barriers, and challenges

Despite promising preclinical results, the clinical translation of single-cell modified probiotics faces significant hurdles that must be systematically addressed.

A primary challenge is the standardization and scalable production of these complex biological products. Probiotics are influenced by various environmental factors during production, processing, storage, and digestion, which affect their survival rate. Unlike conventional drugs, the manufacturing of live bacterial formulations coated with nanomaterials lacks uniform processes. Techniques such as LbL assembly can be precision-dependent and labor-intensive, while the integration of exogenous materials onto bacterial surfaces in a consistent and reproducible manner remains technically challenging. The impact of industrial processes like freeze-drying or spray-drying on the viability and integrity of coated probiotics also requires further elucidation [164].

Additionally, the potential toxicities associated with probiotic modification must be considered. Probiotics applied to patients with severe acute ulcerative colitis are also prone to causing dangerous infections [165]. In a mouse model of extreme severe enteritis induced by a high dose of TNBS, the probiotic *Lactobacillus rhamnosus* can cross the intestinal mucosal barrier and translocate to extra-intestinal organs such as the liver and kidneys [166]. Therefore, for patients with severely damaged intestinal mucosal barriers or severe acute IBD, bacteremia should be guarded against when using probiotics. The potential toxicity of constituent materials poses a significant challenge, including the biopersistence of polydopamine. Its non-degradable nature raises unresolved questions regarding its long-term fate and potential for accumulation [167,168]. Concurrently, the incorporation of metal ions like Fe^3+^ and Zn^2+^ requires careful control to ensure doses remain within safe physiological thresholds, thereby mitigating risks of oxidative stress or other toxicities should the ions be released in an uncontrolled manner [169,170]. Additionally, synthetic polymers like polyethylene glycol (PEG), while effective in shielding probiotics from gastric stress, posing risks of immune reactivity, toxicity or inflammatory responses due to degradation byproducts [171]. Although allergic reactions caused by PEG are extremely rare, with only a few cases reported both domestically and internationally, their symptoms can be exceptionally severe, including cardiac arrest and generalized swelling. These reactions, which may be due to complement activation-related pseudoallergy or IgE-mediated Type I hypersensitivity, are often linked to exposure to significant amounts of PEG [172]. Host immunity and commensal bacteria work together to maintain intestinal balance and prevent pathogen colonization. However, the specific molecular and cellular mechanisms are not yet fully understood. For example, Wang et al. [173] found that in the absence of group 3 innate lymphoid cells (ILC3s), the galactosylation levels of intestinal cells increase, leads to Akk over proliferates, producing succinate which upregulates the expression of virulence factors in pathogens, enhancing their colonization ability and exacerbating host infection. This study not only provides new insights into gut immune regulation but also offers a new perspective for anti-infection treatment, highlighting the safety concerns associated with the use of Akk. Notably, surface modification is used to enhance the gut-targeting capabilities of probiotics, it is crucial to avoid potential safety issues related to excessive colonization. Furthermore, engineered probiotics may trigger unintended immune reactions or horizontal gene transfer [174]. Previous studies have confirmed the biocompatibility and biosafety of coated probiotics in both cellular and animal models. However, it is essential to monitor the dynamic activity and fate of probiotic formulations used for real-world applications.

Furthermore, long-term safety gaps, including risks of microbiome disruption, immune overstimulation, necessitate rigorous longitudinal studies and post-market surveillance. And another point needs to be considered, although surface modification can transiently suppress probiotic metabolism, the net effect on beneficial metabolite production, such as SCFAs, is overwhelmingly positive. This approach represents a paradigm shift from immediate yet vulnerable metabolic activity to a delayed, targeted, and amplified output. The selection of modification method allows for strategic tuning: physical and chemical techniques prioritize the maximal delivery of viable cells for subsequent metabolite production, whereas biological methods enable the direct and sustainable engineering of metabolic output itself. Bridging these gaps is critical to unlocking the full clinical and commercial potential of engineered probiotics in treating chronic diseases. Future research should prioritize investigating how different operational procedures affect commercial coated probiotics, focusing on their impact on cell viability, structural integrity, metabolic activity, and overall probiotic characteristics. It is imperative to gradually improve production standards for commercial coated probiotics. Additionally, extensive preclinical verification is crucial to ensure the biosafety of these probiotics.

### 4.3 Summary and outlook

Single-cell modification probiotics represent a paradigm shift from merely treating symptoms to potentially correcting the underlying biological dysfunction of IBD in a precise, localized, and durable manner. Compared to standard therapies like mesalamine and biologics, this approach offers the potential for superior patient convenience and a fundamentally different, potentially safer mechanism of action by acting as living therapeutics directly within the gut, though clinical proof remains forthcoming. When contrasted with traditional probiotic formulations, these engineered microbes constitute an entirely different category - a true drug with a defined mechanism, as opposed to a supplemental microbial community. For now, this therapeutic class remains a highly promising future direction; the journey from laboratory concept to a safe, approved, and widely available medicine is long and fraught with regulatory and technical challenges, yet the potential to revolutionize the treatment of chronic diseases like IBD makes such innovation one of the most exciting frontiers in medical research today. Tremendous efforts have been devoted to improving the survival and colonization of oral probiotics in the intestine. Surface decoration is a straightforward and frequently used strategy for this purpose. It not only enhances the viability and bioavailability of bacteria but also integrates additional exogenous functionalities. With decoration, sufficient probiotics can reach the intestine and colonize for a longer period, ultimately enhancing their therapeutic effect. A key objective in single-cell surface modification is to develop methods for encapsulating cells within dynamic, degradable shells that protect and regulate them, then can be removed to restore native functionality [175,176]. However, current single-cell modification protocols lack reversible assembly and detachment mechanisms despite their advantages. Multifunctional coatings could combine anti-inflammatory agents like mesalamine with probiotics to enable synergistic mucosal healing and microbial balance. Synthetic biology offers transformative potential through designer probiotics engineered to detect inflammatory biomarkers such as calprotectin, allowing context-responsive drug release to restore homeostasis [34]. Personalizing these approaches, tailoring treatments to individual microbiota and disease profiles, will improve efficacy, minimize side effects, and enhance patient adherence. These innovations aim to shift toward dynamic precision therapies for chronic inflammatory diseases. The future of treating inflammatory conditions involves integrating advances in materials science, synthetic biology, and personalized medicine. Aims to enhance interaction between engineered bacteria and smart material coatings, surpassing current isolated approaches. Key developments include genetically programmed, self-assembling coatings enabling probiotics to autonomously produce and secrete protective matrices, like mussel-inspired adhesive proteins, in response to intestinal signals. Moreover, dynamic materials can be engineered to degrade when exposed to enzymes from the probiotic, ensuring precise activation and release of treatments at the disease site.Interdisciplinary research across fields such as chemistry, physics, materials science, microbiology, and biomedicine is essential to explore a larger number of biocompatible materials to protect living cells. These coating materials need to be improved in terms of encapsulation capability, safety, compatibility with living cells, intestinal adhesion, cost-effectiveness, ease of handling, and stimulus responsiveness. Further research should prioritize investigating the impacts of different operational procedures on commercially coated probiotics, including their effects on cell viability, structural integrity, metabolic activity, and probiotic characteristics. The goal is to establish closed-loop therapeutic systems where probiotics sense inflammatory biomarkers and upregulate surface receptors to recruit drug-loaded nanoparticles, enhancing the therapeutic effect within the lesion. Achieving these advanced systems requires interdisciplinary collaboration across materials science, synthetic biology, and computational modeling. The convergence of synthetic biology programmability with biomimetic materials functionality will advance probiotic therapeutics into autonomous diagnostic and therapeutic platforms capable of adapting to the dynamic pathology of IBD, paving the way for precision medicine.

## 5 Conclusion

This review offers a thorough overview of single-cell modification strategies for probiotics in IBD applications. The strategies are categorized into physical, chemical, and biological methods based on their underlying principles. Single-cell surface modified probiotics represent a paradigm shift in IBD therapy, merging microbiome science with nanotechnology and synthetic biology. These encapsulated probiotics demonstrate improved survival and colonization in the GIT, reduced risk of bacterial translocation, and synergistic effects with nanocoating. This review highlights the transformative potential of surface-modified probiotics, offering hope for safer and more effective IBD therapies based on precision single-cell probiotic modification. It provides a comprehensive summary of recent advancements in probiotic surface modification technologies, analyzing their classification, structural design, and functional attributes while systematically comparing physical, chemical, and biological strategies. Furthermore, it discusses the benefits, limitations, challenges, and future prospects of single-cell probiotic modification. However, challenges persist, and ongoing innovations in material science and genetic engineering promise to unlock the full potential. Future research must prioritize clinical validation, long-term safety assessments, and cost-effective manufacturing to translate these advanced biotherapeutics into mainstream IBD treament.

## CRediT authorship contribution statement

**Li Peng:** Writing – original draft, Project administration, Funding acquisition, Conceptualization. **Xinyu Wang:** Writing – original draft, Visualization, Formal analysis. **Yafen Wang:** Visualization, Validation, Formal analysis. **Jueshuo Guo:** Visualization, Validation, Methodology. **Jianhong Yang:** Writing – review & editing, Funding acquisition, Conceptualization.

## Declaration of competing interest

The authors declare that they have no known competing financial interests or personal relationships that could have appeared to influence the work reported in this paper.

## Data Availability

The data that support the findings of this study are available from the corresponding author upon reasonable request.
